# Leveraging National Germplasm Collections to Determine Significantly Associated Categorical Traits in Crops: Upland and Pima Cotton as a Case Study

**DOI:** 10.3389/fpls.2022.837038

**Published:** 2022-04-26

**Authors:** Daniel Restrepo-Montoya, Amanda M. Hulse-Kemp, Jodi A. Scheffler, Candace H. Haigler, Lori L. Hinze, Janna Love, Richard G. Percy, Don C. Jones, James Frelichowski

**Affiliations:** ^1^Department of Crop and Soil Sciences, North Carolina State University, Raleigh, NC, United States; ^2^Genomics and Bioinformatics Research Unit, United States Department of Agriculture - Agricultural Research Service (USDA-ARS), Raleigh, NC, United States; ^3^Crop Genetics Research Unit, United States Department of Agriculture - Agricultural Research Service (USDA-ARS), Stoneville, MS, United States; ^4^Department of Plant and Microbial Biology, North Carolina State University, Raleigh, NC, United States; ^5^Crop Germplasm Research Unit, United States Department of Agriculture - Agricultural Research Service (USDA-ARS), College Station, TX, United States; ^6^Cotton Incorporated, Raleigh, NC, United States

**Keywords:** trait association, categorical data, cotton, crop germplasm, breeding

## Abstract

Observable qualitative traits are relatively stable across environments and are commonly used to evaluate crop genetic diversity. Recently, molecular markers have largely superseded describing phenotypes in diversity surveys. However, qualitative descriptors are useful in cataloging germplasm collections and for describing new germplasm in patents, publications, and/or the Plant Variety Protection (PVP) system. This research focused on the comparative analysis of standardized cotton traits as represented within the National Cotton Germplasm Collection (NCGC). The cotton traits are named by ‘descriptors’ that have non-numerical sub-categories (descriptor states) reflecting the details of how each trait manifests or is absent in the plant. We statistically assessed selected accessions from three major groups of *Gossypium* as defined by the NCGC curator: (1) “Stoneville accessions (SA),” containing mainly Upland cotton (*Gossypium hirsutum*) cultivars; (2) “Texas accessions (TEX),” containing mainly *G. hirsutum* landraces; and (3) *Gossypium barbadense* (Gb), containing cultivars or landraces of Pima cotton (*Gossypium barbadense*). For 33 cotton descriptors we: (a) revealed distributions of character states for each descriptor within each group; (b) analyzed bivariate associations between paired descriptors; and (c) clustered accessions based on their descriptors. The fewest significant associations between descriptors occurred in the SA dataset, likely reflecting extensive breeding for cultivar development. In contrast, the TEX and Gb datasets showed a higher number of significant associations between descriptors, likely correlating with less impact from breeding efforts. Three significant bivariate associations were identified for all three groups, *bract nectaries:boll nectaries*, *leaf hair:stem hair*, and *lint color:seed fuzz color*. Unsupervised clustering analysis recapitulated the species labels for about 97% of the accessions. Unexpected clustering results indicated accessions that may benefit from potential further investigation. In the future, the significant associations between standardized descriptors can be used by curators to determine whether new exotic/unusual accessions most closely resemble Upland or Pima cotton. In addition, the study shows how existing descriptors for large germplasm datasets can be useful to inform downstream goals in breeding and research, such as identifying rare individuals with specific trait combinations and targeting breakdown of remaining trait associations through breeding, thus demonstrating the utility of the analytical methods employed in categorizing germplasm diversity within the collection.

## Introduction

Global agriculture production is facing major challenges, including demands to increase crop productivity and quality while sufficiently preserving natural ecosystems, addressing climate change and tolerance of intense weather events, increasing agricultural resource use efficiency, and enhancing biotic and abiotic stress resistance (FAO, 2017; Tian et al., 2021). To address these challenges, a constant interaction between plant breeding and fundamental research is needed, and both approaches have been used to address challenges of crop production for food, fiber, fuel, animal feeds, and ornamental uses, among others (Gillespie and van den Bold, 2017; Ramankutty et al., 2018; Zhao et al., 2019; Nguyen and Norton, 2020). Particularly, in the 21st century, agricultural intensification has relied on producing crops with genetic uniformity. Although these practices have benefits, they potentially increase crop susceptibility to pests, diseases, and environmental stress. To overcome those issues, the worldwide germplasm collections are essential to collecting and conserving living plant material, solving agricultural production problems, as well as conserving plant genetic diversity for future needs (Börner and Khlestkina, 2019; Nguyen and Norton, 2020). Among them, the largest collection in the world is the United States National Plant Germplasm System (NPGS) maintained by the United States Department of Agriculture - Agricultural Research Service (USDA-ARS). In the 1970’s and 80’s, the USDA mandated conservation of historical cultivars and crop wild relative germplasm for agricultural security (Wilkes and Williams, 2008). The NPGS is charged to acquire, conserve, document, distribute, evaluate, and characterize crop germplasm in order to safeguard the genetic diversity of agriculturally important plants (Allender, 2011; Byrne et al., 2018). Permanent collections and curators were established and available or acquired germplasm was re-routed to be first handled by the curators then maintained and distributed to users. There are currently 44 crop germplasm collections in the NPGS, the majority of which collect data on observable qualitative traits for each accession in the collections, including the National Cotton Germplasm Collection (NCGC) for *Gossypium* species (Postman et al., 2010; White et al., 2011).

Cotton is one of the most important cash crops around the world, and it provides the largest renewable source of fiber in addition to edible oil and protein (Campbell et al., 2010; Ahmad and Hasanuzzaman, 2020; Kumar et al., 2021). The NCGC began in 1989 and is physically maintained in College Station, TX, United States. It currently includes about 50 species of *Gossypium* and 10,459 total cotton accessions^1^. The collection is accompanied by information on the species classification and historical context of accessions, as traditionally described by a curator in the USDA-ARS Crop Germplasm Research Unit (CGRU). The NCGC primarily contains *G. hirsutum* and *G. barbadense*, which are the two main cultivated tetraploid cotton species (the other two cultivated types are diploids) (Grover et al., 2014). Upland cotton (*G. hirsutum –* Gh) and Pima cotton (*G. barbadense* – Gb), represent 75% of the total number of accessions in the NCGC collection. The Gh collection contains two main subsets as follows. (1) The Stoneville accessions (SA) mainly represent obsolete Gh cultivars originally collected at the Mississippi State University Delta Branch Experiment Station in Stoneville, Mississippi. (2) The Texas accessions (TEX) include photoperiodic landraces (i.e., primitive domesticated germplasm) or tropical materials as originally housed at Texas A&M University, College Station, Texas. The Pima accessions (Gb) were initially curated in Phoenix, Arizona, and the current group may contain a mix of landraces and cultivars, although specific subset information is not available (Percy et al., 2014).

In order to better characterize the diversity in the NCGC, a rating scale was established in 2006 for 36 phenotypic descriptors that encompass the diversity across *Gossypium* species in the collection, as observed by researchers in the CGRU (Yuan et al., 2021). For the past decade, the NCGC standardized and expanded descriptors to cover the consolidated sub-collection accessions and *Gossypium* species. However, the early stages of the cotton germplasm collection were sub-collections in different locations so historical descriptors and ratings differ. This systematic approach for describing traits has been used for evaluating many of the accessions in the NCGC over the last 11 years in the field in three different locations: (1) College Station, Texas; (2) Tecomán, Colima, Mexico; and (3) Liberia, Guanacaste, Costa Rica (Percy and Kohel, 1999; Wallace et al., 2008; Frelichowski and Percy, 2015; Yuan et al., 2021). Each of the 36 descriptors has a rating scale with a discrete number of non-numerical categories, or descriptor states, which encompass the variation in individual cotton accessions. Stated in another way, the rating scale for each trait contains a set number of categories or categorical variables, which may include for example presence, absence, and intermediary states of the trait between presence and absence (Percy et al., 2014; UPOV-Council, 2019; Cerda and Varoquaux, 2020).

**FIGURE 1:**
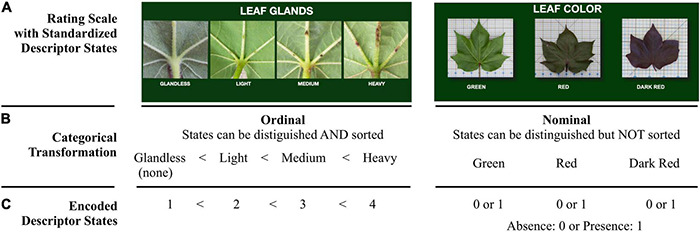
Example of a categorical classification and encoding transformation of phenotypic descriptors. **(A)** Rating scales showing four (for leaf glands) or three (for leaf color) standardized descriptor states. **(B)** Examples of classifying two types of descriptor states, ordinal and nominal. **(C)** Encoding data transformation from text format to integer format prior to applying statistical analysis.

Two of the cotton descriptors are illustrated in Figure 1A, leaf glands and leaf color. The rating scale for leaf glands has four descriptor states: glandless, light, medium, or heavy. The leaf glands descriptor is ordinal because there is a natural order within the range, but the distances between the states are not known. The rating scale for leaf color has three states: green, red, or dark red. The leaf color descriptor is nominal because its states are recognizable, but they lack inherent order. Neither nominal or ordinal variables have true quantitative values, but they can be evaluated through categorical analysis after grouping into a set of mutually exclusive unordered (nominal) or ordered (ordinal) categories (Watson, 2014; UPOV-Council, 2019) (Figure 1B). Classification of descriptor states into nominal and ordinal data types allows for the transformation of the data into a large matrix, and this, in turn, supports the use of statistical methods including bivariate association analysis to further characterize the large data set (Figure 1C). Bivariate association analysis determines whether or not there is a statistically significant relationship between any two descriptors within each group analyzed. Two descriptors are significantly associated if one of them tends to display specific states when the state of the other descriptor changes. Conversely, there is no significant association between two descriptors if their states change independently of each other (Watson, 2014; UPOV-Council, 2019). The evaluation and analysis of categorical traits have been previously suggested by the International Union for the Protection of New Varieties of Plants (UPOV-Council, 2019) as a means of demonstrating distinctness or statistically significant grouping patterns of different plant varieties.

Some examples of how categorical traits matter for cotton improvement are described below. The red color of cotton bolls, bracts, leaves, and stems may be useful for separating cotton genotypes during field tests, and it may also indicate enhanced resistance of red accessions to certain insects and/or pathogens (Long et al., 2019; Zhang et al., 2019). Likewise, the presence/absence of lysigenous glands, which contain terpenoid aldehydes including sesquiterpenoid gossypol, on bolls, leaves, and stems affects the degree of natural protection against insects. Conversely, the toxicity of these compounds to non-ruminant animals and humans limits the uses of cotton seeds and plant parts (Cai et al., 2010), which implies that breeders may want to alter the number and/or distribution of the glands (Zhou et al., 2013; Park et al., 2019; Gao et al., 2020). Other traits such as nectar glands on bolls, bracts, and leaves can, in an ecological context, provide nutrition for insects and microorganisms, while they promote insect damage in a crop context (Park et al., 2019). Moreover, the presence of hairs on leaves and stems may contribute to resistance to certain insects (i.e., Jassids) (Knight, 1952).

If meaningfully compared, the standardized phenotypic descriptors can be integrated with other phenotypic and genotypic data reported by NCGC to extract hidden information and expand the utility of the germplasm collection. We describe statistical methods to evaluate and extract additional meaning from phenotypic descriptors collected by a germplasm team. We leveraged a decade of collected data to compare descriptors within three major groups of *Gossypium* accessions maintained in the NCGC, including Pima cotton (Gb group) and cultivated Upland cotton (SA group) and its less-improved relatives (TEX group) (*G. hirsutum*). In this analysis, we add value to three of these sub-collections by identifying accessions that do have complete records for standardized phenotype descriptors and then exploring the descriptors. The results reveal: (a) distributions of character states for each descriptor within each of the three groups; (b) statistically significant bivariate associations between paired descriptors within each group; (c) label-blind, descriptor-based, clusters of accessions within a species; and (d) the ability to utilize clustering of descriptor data to identity the species of an accession. We anticipate that our prototypical analysis for cotton will be adaptable to the germplasm collections of other crops.

## Materials and Methods

### Phenotypic Data and Accession Identification

A categorical analysis was applied using the phenotypic descriptors for selected accessions publicly available in the NCGC, which is part of the Germplasm Resource Information Network (GRIN)-Global (Cotton – see Text Footnote 1). The definition of the categorical descriptor scoring and methods for collecting the scores are reported in Supplementary Data 1. The scores correspond to standardized states for each descriptor that subdivide the overall range of the phenotype as observed in *Gossypium*. A particular descriptor may also include the “absent” state. The standardized descriptors and their rating scales are shown in table format on the CottonGen research community database website^2^ (see also Supplementary Table 1 – 05/01/2021), this is constantly updated as traits are added for evaluation.

**FIGURE 2:**
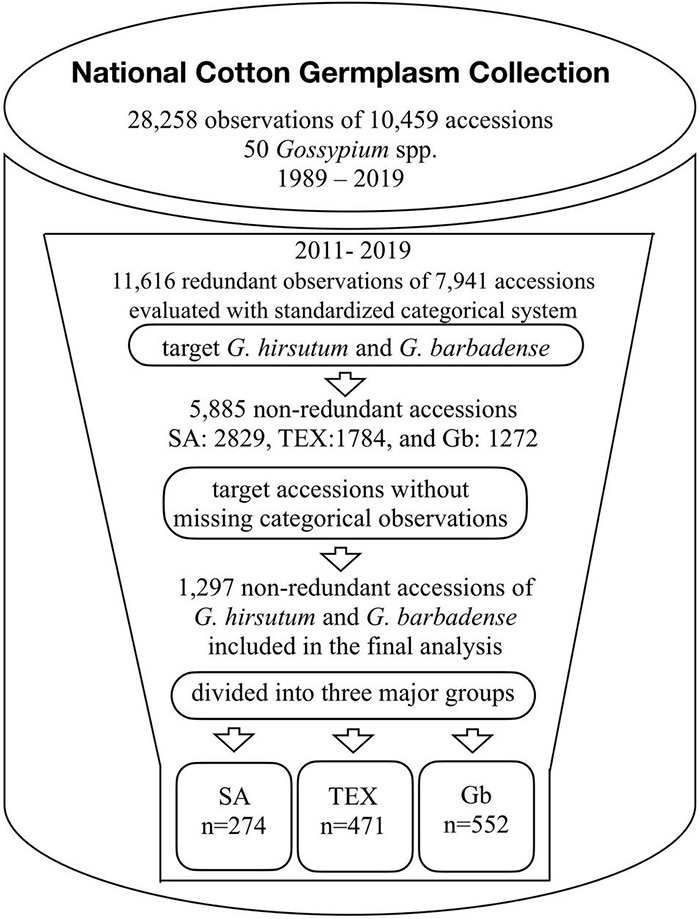
Summary of the workflow leading to the final categorical trait descriptor dataset. Redundant observations reflect multiple evaluations of a single accession in multiple locations and/or different years. The data collected from 2011 to 2019 contained 11,616 field observations of approximately 21 *Gossypium* spp. Our work focused on data subsets enriched in *G. hirsutum* (Gh) and *G. barbadense* (Gb) accessions: SA, mainly Upland cotton cultivars; TEX, mainly photoperiodic/tropical landraces; and Gb – mainly Pima cotton. Numbers presented are as of May 2019, data is added to the database as it is collected so numbers are always increasing.

The categorical descriptors for selected cotton accessions were obtained from the GRIN-Global system^3^. In the history of NCGC, a total of 28,258 observations on 10,459 accessions of 50 *Gossypium* species were made in the field between 1989 and 2019 (Figure 2). We studied data collected between 2011 and 2019 in correspondence with the time that observations began for 36 standardized descriptors under the direction of the USDA-ARS Crop Germplasm Research Unit (Wallace et al., 2008; Percy et al., 2014). In this last decade, a total of 11,616 observations (41% of the total set) on 7,941 unique accessions (as of May 2019) were in the database, but testing some of the accessions in multiple years and/or locations resulted in redundant records. Some of the records also had missing data points for one or more of the 36 descriptors. In order to obtain non-redundant and complete records, the dataset was filtered using the following criteria. (1) Only accessions that belong to the SA, TEX, and Gb groups were selected. (2) The accessions with redundant records were randomly processed to select only one observation set per accession. (3) The accessions with missing information for any of the 36 descriptors were removed. After this filtering process, a total of 1,297 accessions with complete records were identified (SA, 274; TEX, 471; and Gb, 552, Figure 2). The accession IDs, the number of total seed requests per accession since 2007, and the associated descriptor information can be found in Supplementary Tables 2–4, for the SA, TEX, and Gb groups, respectively. The analysis finally included 46,692 data points.

### Phenotypic Distributions and Data Transformations

For further analysis, 33 of 36 descriptors were retained because they were expected to be independent of the environment. Specifically, the scores for maturity, photoperiodic rating, and productivity were removed. The number of accessions in each group displaying each state of the analyzed descriptors was determined and displayed in distribution plots showing the observed variation across groups (Supplementary Figure 1). For statistical purposes, the descriptors were classified as nominal or ordinal prior to performing data transformations on the categorical scoring data of the remaining 33 descriptors (Figure 1C and Supplementary Table 5). All notations of segregation (seg)/off-type (i.e., where an accession was found to have varying levels of a descriptor) were removed from the analysis because the related phenotype was too complex or diverse to fit into the standardized rating scale for that descriptor (Supplementary Data 1). Only descriptors with two or more states observed in the field could be included in statistical analysis. To generate reasonable statistical power, each descriptor state was required to be represented by 5 or more accessions within the final data matrix. According to standard practice (Cochran, 1954; Camilli and Hopkins, 1978), some of the descriptor states were removed or combined if two or more of them together would include at least 5 observations (Supplementary Table 6). The changed instances were less than 5% of the initial data set that was used to plot phenotype distributions. This procedure explains why some descriptor states in the distribution plots are not also seen in the mosaic plots.

### Bivariate Association and Contingency Analysis

The encoding transformation of the 33 descriptors produced a final data matrix for each group (SA, TEX, and Gb), which was then used for bivariate association analysis in JMP Pro 15.2.0 software (SAS Institute Inc., Cary, NC, United States). A contingency table was generated based on the comparison of each possible pair of descriptors. These tables show the number of observations for all of the different combinations of states of each descriptor. The contingency tables reveal how the states of descriptor 1 are contingent on the states of descriptor 2. We chose alpha = 0.01 as the standard for assessing significance. *P*-values were calculated by either Fisher’s exact test (if both descriptors had only two states) or the Chi-square independent test (if at least one of the descriptors being compared had more than two states). The initial *p*-values were obtained as a list where each value corresponded to an independent bivariate association. Then the list was converted into a square matrix prior to adjusting for the False Discovery Rate (FDR) (Benjamini and Hochberg, 1995; Chiu, 2002). The FDR was only applied to the lower triangular matrix in order to avoid double-counting of the same comparison. The resulting FDR-corrected p-values of the bivariate associations were visualized in a heatmap for each of the three major groups of accessions. For each position in the heatmap, an associated mosaic plot shows a graphical representation of the two-way frequency table produced by the contingency analysis^4^.

### Unsupervised Analysis: Clustering Analysis and Multivariate Procedure

The same data set used for bivariate association analysis was used for unsupervised clustering analysis. K-modes clustering was used to explore similarities and/or differences among the three groups (see Supplementary Tables 2–4). The data matrix inclusive of all three groups was transformed using the scikit-learn 0.24.2 software (Pedregosa et al., 2011) into levels reflecting the rating scales of each descriptor prior to clustering analysis. K-modes unsupervised clustering analysis was run using kmodes version 0.11.0 (de Vos, 2021). The clustering analysis assumes a fixed number of clusters and tries to maximize the homogeneity within the clusters, so the analysis was run with *k* = 2 (aiming to discriminate Gh and Gb accessions) and *k* = 3 (aiming to discriminate SA, TEX, and Gb groups). The analysis depended on the prior encoding of the descriptors as nominal/ordinal, and the clustering was blind to NCGC labels for accession species/groups. The correlation of results of the algorithm species/group placement with the NCGC species/group labels was evaluated. Results were also evaluated by calculating an accuracy score of clustering using silhouette scores with the scikit-learn 0.24.2 software. The script implemented for this analysis is reported in Supplementary Data 2 and the input file to run this analysis is reported in Supplementary Table 7.

An unsupervised multivariate procedure known as Multiple Correspondence Analysis (MCA) was also used to explore the relationship of the SA, TEX, and Gb accessions (Abdi and Valentin, 2007). In this procedure dimensionality reduction is applied over the categorical descriptors then identification of the non-linear interactions is performed. Afterward the first components are used to visualize the MCA “cloud of individuals” or the similarity structure of the accessions (see Supplementary Tables 2–4) (Kassambara, 2016; Nguyen and Holmes, 2019). The MCA analysis was applied in R, using the library FactoMineR version 2.4 (Lê et al., 2008) and the visualization was obtained using the library factoextra version 1.0.7 (Kassambra, 2022). The script implemented for this analysis is reported in Supplementary Data 3.

### Bivariate Association Analysis Using Unsupervised Clustering Result

Three out of the nine sets identified by the unsupervised clustering analysis were reanalyzed using the bivariate association approach. The TEX accessions which clustered as TEX (*n* = 308), the SA accessions which clustered as TEX (*n* = 156) and the SA accessions which clustered as SA (*n* = 251) were processed (Supplementary Table 9). The remaining sets were not evaluated due to the low number of accessions clustered except for Gb, which largely contained the same set of samples as the prior bivariate association analysis.

### Multiple Correspondence Analysis to Extract Information Content of Descriptors

The categorical traits were evaluated with the same MCA strategy as above (di Franco, 2016) to identify the contribution and correlation between descriptors. The contribution of each descriptor identified how much influence each categorical trait had in determining the overall information content relative to the entire set of traits (di Franco, 2016). The relationship between each of the variables was represented by calculating the correlation ratios between the accession coordinates on one component and each of the categorical variables, these results were visualized as the MCA “cloud of variables,” or the similarity structure of categorical traits (Husson et al., 2010).

## Results

### Cotton Accessions and Distributions of Descriptor States

**FIGURE 3:**
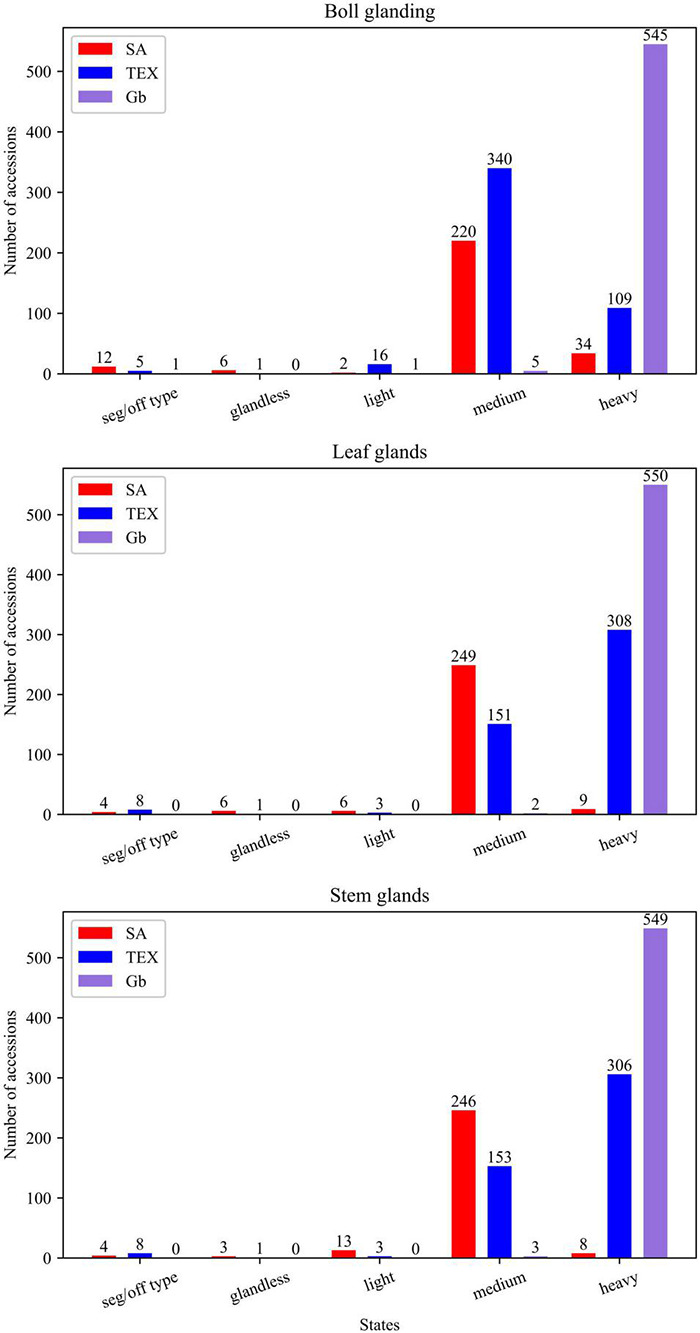
Distribution of gland-related descriptors across plant structures and species. Seg/off type: segregation-off type condition, in which an accession was not homogeneous for a single state.

The 33 cotton descriptors analyzed represent attributes evaluating vegetative, reproductive, and architectural structures of the plant for the three groups of accessions (SA, TEX, and Gb). Features such as color, nectaries, shape, or glands may be defined for multiple parts or aspects of the plant, with the different occurrences then counted as separate descriptors (Table 1). The three cotton groups analyzed often showed different patterns of variation for the states of each descriptor. There were cases where descriptor states were uniform in one group, but showed diverse distribution in others, and instances where each group displayed a different range of states for a particular descriptor. For example, glands are distributed across multiple parts of the plant and are, therefore, evaluated in bolls, leaves, and stems. The distributions within the different tissues showed that most SA and TEX accessions are medium or heavy glanded, whereas the Gb accessions were almost uniformly heavy glanded across all parts of the plant (Figure 3). Distributions for all descriptors in the three cotton groups analyzed are reported in Supplementary Figure 1.

**TABLE 1:** Summary of 33 phenotypic descriptors analyzed in this study. Each descriptor, as marked by x, reflects a combination of a feature and the plant structure where the feature was evaluated.

Plant structure															
		Boll	Bract	Canopy	Fruiting	Growth	Leaf	Lint	Petal	Pollen	Seed fuzz	Seed	Stem	Locule	Stigma
**Feature**	Color	x	x				x	x	x	x	x		x		
	Nectaries	x	x				x								
	Shape	x					x								
	Type		x	x	x							x			x
	Habit					x									
	Glands	x					x						x		
	Pitting	x													
	Pointing	x													
	Size	x					x								
	Teeth number		x												
	Teeth size		x												
	Hairs						x						x		
	Number													x	
	Spot								x						
	Density										x				

*Supporting document including the descriptor definitions (Supplementary Data 1).*

### Bivariate Associations of the Phenotypic Descriptors in Stoneville Accessions, Texas Accessions, and *Gossypium barbadense*

**FIGURE 4:**
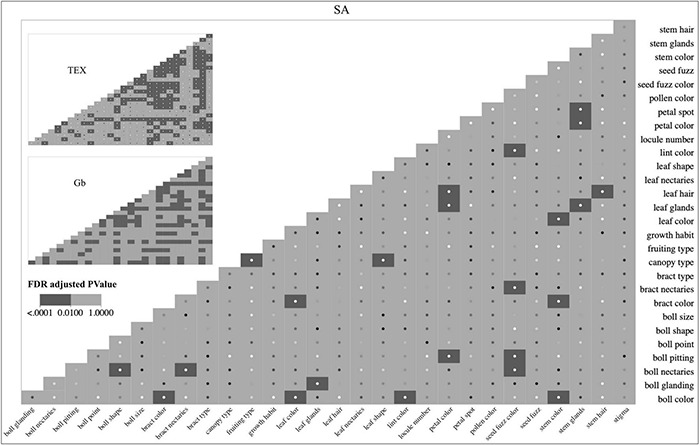
Heat maps of the bivariate descriptor associations were independently evaluated for the SA, TEX, and Gb groups. Larger versions of the TEX and Gb heat figures are in Supplementary Figures 2, 3. Interactive heat maps linked to the contingency tables and mosaic plots for each association evaluated are available on-line (https://usda-ars-gbru.github.io/categorical_analysis_cotton/). Dark gray boxes indicate *p* < 0.01. The sample size of accessions for each group is SA: 274, TEX: 471, and Gb: 552.

While plotting distributions of the states of individual descriptors across groups can be informative, it is also useful to identify cases where significant associations between descriptors occur within a group through bivariate association analysis. As an example, a breeder could ask the question: do the descriptor states of leaf glands change in parallel with the descriptor states of leaf hairs in different groups of *Gossypium* accessions? Figure 4 shows heat maps displaying the significant ‘*descriptor_1:descriptor_2’* associations for the SA, TEX, and Gb groups independently. They show that the ‘*leaf glands:leaf hair’* comparison is significant for SA and TEX (*p* ≤ 0.01). As previously mentioned, the association could not be analyzed in Gb because all of the accessions were heavy-glanded. Therefore, the ‘leaf glands’ descriptor does not appear in the Gb heat map.

From an overall perspective, the SA group had 23 significant associations out of 406 tested (all possible pairwise comparisons). The 23/406 ratio for SA (5.6%) compares to 153/406 for TEX (37.6%) and 122/351 for Gb (34.7%) (Figure 4). Among the three groups evaluated, most of the categorical descriptors show at least one significant association with another descriptor. The SA group had the largest number (9) of categorical descriptors with no significant association, meaning that its states changed independently of any other descriptor (stigma, seed fuzz, pollen color, locule number, leaf nectaries, growth habit, bract type, boll size, and boll point). Comparatively, all descriptors in TEX were significantly associated with at least one other descriptor, and Gb was similar with only one descriptor (boll point) lacking at least one association (Figure 4).

**FIGURE 5:**
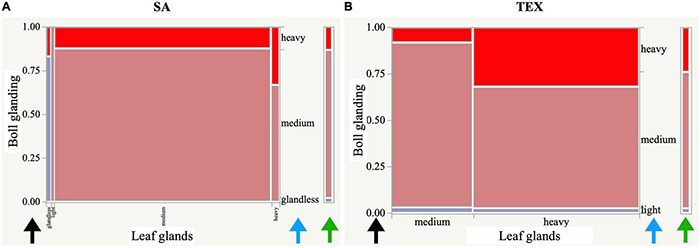
Mosaic plots displaying the degrees of glanding in leaves versus bolls. Plots are shown for **(A)** the SA and **(B)** TEX groups. The plots are divided into rectangles as a stacked bar chart so that the vertical length of each rectangle reflects the proportion of the *Y* variable in each state (blue arrows) of the *X* variable and is a graphical representation of a contingency table. The scale of the vertical axis at left on each plot shows the response probability (black arrows). The whole axis is equivalent to a probability of one, representing the total sample. Fill colors are showing boxes reflecting the phenotype on the *Y*-axis with legend to the right of the figure (green arrows).

Examples of the contingency analysis are presented as mosaic plots, or stacked bar charts (Figure 5), which facilitate visual comparison of results between the groups analyzed. This type of plot was possible in cases where the descriptor had more than one state reported within the rating scale. These plots are important to analyze in cases of two or more of the cotton groups having the same significant ‘*descriptor_1:descriptor_2’* association, because the co-varying descriptor states may or may not be the same between groups (as shown here between Figures 5A,B). In each mosaic plot, the horizontal (*X-*) axis shows the states of descriptor_1 that were present in the group, with the width of each corresponding column portraying the proportion of accessions observed with that state of descriptor_1. The double vertical (*Y-*) axes together (black and blue arrows) describe descriptor_2, the vertical length of the bars is proportional to the number of accessions with each state of descriptor_2. The left-side *Y*-axis pertains to the proportion of descriptor_2 states found within the *X*-axis descriptor_1 variable states providing the overall likelihood that a trait state will be observed with the *X*-axis descriptor_1 trait state. The right-side *Y*-axis outlines the overall proportions of descriptor_2 (green arrow^5^). Figure 5 shows the ‘*leaf glands:boll glanding’* mosaic plots for SA and TEX. In the SA group, most accessions had glands on bolls and leaves, and a medium state of glanding dominated in both organs. Among rare accessions with glandless leaves, about 80% also had glandless bolls. On the contrary, the TEX group contained numerous accessions with heavy glanding in leaves and bolls, and no glandless associations were present using the baseline criteria of this study (Figure 5).

### Relationships Between the Significant Descriptor Associations Existing in Stoneville Accessions, Texas Accessions, and *Gossypium barbadense*

**FIGURE 6:**
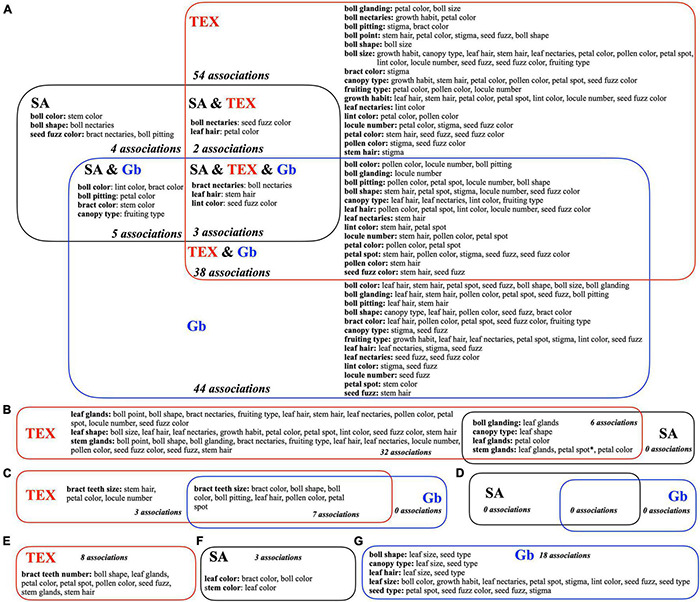
Venn diagrams showing high confidence bivariate descriptor associations within and among three groups of *Gossypium* accessions, SA, TEX, and Gb. Bivariate significance is based on the FDR adjusted *P*-values. Each sector is labeled with the relevant group name(s) and the number of associations it contains. Descriptors within a sector that had high confidence associations (*P* < 0.01) are listed in bold and alphabetical order, with the associated descriptors following in plain text. For example, in the SA and Gb sector **(A)**, the first line indicates two high confidence bivariate associations: boll color with (1) lint color and boll color with (2) bract color. Descriptors listed in plain text may occur in more than a single bold category, but each bivariate descriptor-to-descriptor combination should occur only once in the whole diagram. If an association between two descriptors does not appear, the *p*-value was >0.01 for that comparison. **(A)** Significant categorical descriptors evaluated between the three groups. **(B)** TEX vs. SA. **(C)** TEX vs. Gb. **(D)** SA vs. Gb. **(E)** Only TEX. **(F)** Only SA. **(G)** Only Gb. *In **(B)**, the TEX and SA *stem glands:petal spot* association is the only case where the states of the descriptor states was independently modified for each group (modification shown in Supplementary Table 6); results are statistically significant but the descriptor states had different states in the TEX and SA group.

The significant descriptor associations within each separate group (Figure 4) were intersected to identify commonalities and differences between the three groups, when possible, as shown in the Venn diagram (Figure 6). Most ‘*descriptor_1:descriptor_2’* evaluations were performed in all three groups (Figure 6A), but some descriptors were not analyzed in this way because they had the same state (homogeneous) in more than 98% of the accessions of one or more groups. These predominant homogeneous state phenotypes in each group were: for SA, leaf size (medium), seed type (free), bract teeth size (large), and bract teeth number (medium); for TEX, leaf color (green), leaf size (medium), seed type (free), and bract type (normal); and for Gb, leaf color (green), leaf shape (normal), stem glands (heavy), leaf glands (heavy), bract type (normal), and bract teeth number (medium) (Supplementary Figure 1). Of these predominant phenotypes, none are shared across all three groups, but three pairs are shared across two groups: SA and Gb both have medium bract teeth number and TEX and SA both have medium leaf size and the free seed type. In some other cases, a descriptor lacked multiple states in all three groups, which implied that only one or two groups could be compared. Correspondingly, the diagram in Figure 6 is divided into sections showing comparisons between all three groups (SA vs. TEX vs. Gb, Figure 6A), across two groups (SA vs. TEX, TEX vs. Gb, or SA vs. Gb, Figures 6B–D) or only in one group (Figures 6E–G).

**FIGURE 7:**
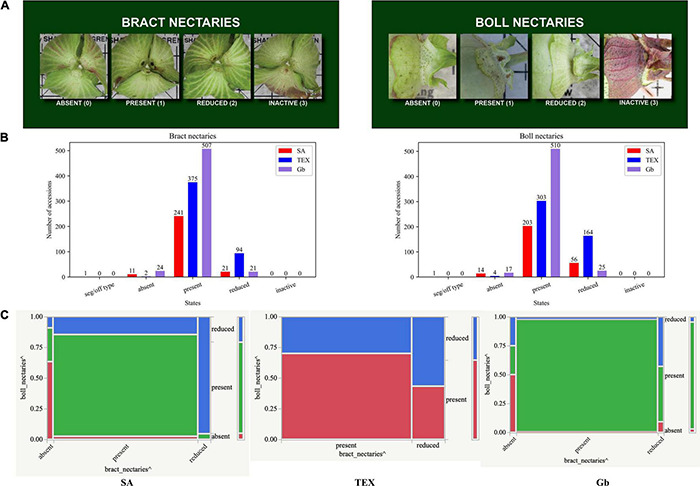
Information related to boll and bract nectaries in SA, TEX, and Gb. The results independently showed a significant association among the three groups (FDR adjusted *P*-values – *P* < 0.01). **(A)** The photos show the rating scale for bract and boll nectaries. **(B)** The two nectary descriptors show a similar distribution of state. (States with ≤5 observations and the seg/off type category were removed prior to bivariate association analysis). **(C)** Mosaic plots from contingency analysis show the relationship among descriptor states for each group evaluated. See Figure 5 for details on the mosaic plot interpretation.

Across the three-group comparison, there were only three shared associations: ‘*bract nectaries:boll nectaries*’, ‘*leaf hair:stem hair*’, and ‘*lint color:seed fuzz color*’ (Figure 6A). Breeders are concerned about nectaries due to their role in attracting insects, which often act as pests during production of cotton, given its capacity for self-pollination (Rudgers et al., 2004; Frelichowski and Percy, 2015; Zeng et al., 2018; Park et al., 2021). Here we use the names for nectary states as shown in Figure 7A. Both types of nectaries were ‘present’ in the majority of accessions analyzed for all three groups (Figure 7B). However, analysis of the mosaic plots shows some differences between the associated states of each descriptor between groups (Figure 7C). In the SA group, about 80% of the accessions had ‘present’ bract nectaries. Of these, about 80% also had boll nectaries. For the minority of accessions with reduced bract nectaries, about 95% of them also had reduced boll nectaries. Among the rare SA accessions that lacked bract nectaries, about 60% of them also lacked boll nectaries. However, for TEX, about 70% of the accessions had ‘present’ bract and boll nectaries. Of the remaining 30% with reduced bract nectaries, the boll nectaries were either ‘present’ or reduced in an approximately 50:50 ratio. Finally, the nectary traits in Gb were most similar to SA, but ‘present’ bract and boll nectaries existed in 99% of the accessions. When Gb bract nectaries were reduced in rare accessions, boll nectaries were either ‘present’ or reduced in an approximately 50:50 ratio (Figure 7C). The other two pairs of descriptor associations that were consistently found among the three accession groups (‘*leaf hair:stem hair*’, and ‘*lint color:seed fuzz color*’) are further illustrated in Supplementary Figures 4, 5, respectively.

Other bivariate descriptor associations were shared between only two groups or found in only one group. Between the SA and the TEX groups, six consistent associations were identified, including two descriptor pairs related to gossypol glands, ‘*boll glanding:leaf glands’* and ‘*stem glands:leaf glands’* (Figure 6B). Between the TEX and the Gb groups, seven diverse plant descriptors were consistently associated with bract teeth size (Figure 6C). No high confidence associations were identified in the SA to Gb comparison (Figure 6D). Finally, some significant associations occurred only in one group (Figures 6E–G).

### Unsupervised Clustering Analysis Across Species

Unsupervised clustering analysis was first based on the combined set of SA plus TEX groups (745 total accessions) and the Gb group (552 total accessions) that we had selected for analysis from NCGC in order to determine if the method would generate two species-enriched groups (*k* = 2). In general, the method worked well: the unsupervised (i.e., blind to the NCGC label) k-modes analysis clustered 97.2% of SA plus TEX accessions together and 98.9% of the Gb accessions together (Cluster 2.1 and Cluster 2.2, respectively, in Table 2). Only 6 accessions originally labeled as Gb (0.8%) were clustered with the Gh set and only 21 accessions originally labeled as Gh (3.8%) were classified as Gb (Table 2). See Supplementary Table 8 for accession IDs.

**TABLE 2:** Summary of K-modes unsupervised clustering (*k* = 2).

	Unsupervised clustering sets	
Original group labels	Cluster 2.1 (Gh)	Cluster 2.2 (Gb)
SA plus TEX	724	21
Gb	6	546

*Two-cluster analysis was designed to group accessions by species. Clusters were arbitrarily numbered based on k-value and unique ID, i.e., ‘Cluster 2.1’, and the species identifier was assigned afterward based on the majority of pre-labeled accessions in NCGC that it contained.*

*Silhouette score: 0.33. Accessions analyzed were: SA plus TEX, 745 accessions; and Gb, 552 accessions. The Accessions IDs are reported in Supplementary Table 8.*

### Unsupervised Clustering Analysis Across Groups

Unsupervised clustering analysis was then based on a combination of all three groups under analysis to determine if the method would separate SA and TEX accessions into two groups while also clustering Gb into a third group (*k* = 3). The number of accessions analyzed were: 471 for TEX; 274 for SA; and 552 for Gb (see Supplementary Table 9 for Accessions IDs and clusters.). Results of the clustering are shown in Table 3. For the SA group, 91.6% of the accessions were clustered together (Cluster 3.2) and most of the remaining accessions were clustered with the TEX group (Cluster 3.1). A lesser percentage (65.4%) of the TEX group clustered together (Cluster 3.1), with the others (33.1%) grouping with the SA set (Cluster 3.2). Finally, 98.3% of the Gb group clustered together (Cluster 3.3), with a few (1.3%) of the accessions originally labeled as Gb clustering with the TEX group (Cluster 3.1) (Table 3).

**TABLE 3:** Summary of K-modes unsupervised clustering (*k* = 3).

	Unsupervised clustering sets		
Original group labels	Cluster 3.1 (TEX)	Cluster 3.2 (SA)	Cluster 3.3 (Gb)
TEX	308	156	7
SA	21	251	2
Gb	9	0	543

*Three-cluster analysis was designed to test for separation between all three groups of accessions. Clusters were arbitrarily numbered based on k-value and unique ID, i.e., ‘Cluster 3.1’ and the group identifier was assigned afterward based on the majority of pre-labeled accessions in NCGC that it contained.*

*Silhouette score: 0.22. The Accessions IDs and clusters are reported in Supplementary Table 9.*

### Unsupervised Multiple Correspondence Analysis – Clustering Individuals

**FIGURE 8:**
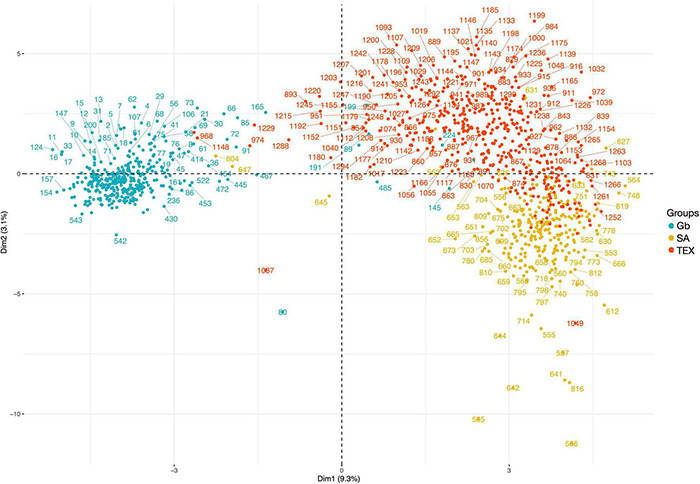
Multiple correspondence analysis cloud of individuals across groups. The graph shows similarities of the individuals in terms of all categorial descriptor variables. The first two principal components are shown (DIM 1 + DIM 2: 12.4%). Accessions are colored by group for Gb (blue), SA (yellow), and TEX (red); accession index numbers can be found in Supplementary Tables 2–4.

The MCA “cloud of individuals” appeared to provide similar results to the unsupervised clustering analysis across groups. In the cloud there are 2 notable groups – 1 composed of mostly Gb and 1 composed of SA and TEX accessions. On one hand, most of the Gb accessions are in the negative area between Dim 1 and 2. On the other hand, the SA-TEX cloud shows that most of the SA accessions are in the bottom right area and the TEX are in the top right, though there is a group of TEX accessions located in the SA area (Figure 8).

### Bivariate Associations Based on Clustering

Three out of the nine sets identified by the unsupervised clustering analysis (*k* = 3) were reanalyzed using the bivariate association approach. In cluster 3.1, the TEX accessions clustered as TEX (n:308); and for Cluster 3.2, the SA accessions clustered as TEX (*n* = 156) and the SA accessions clustered as SA (*n*:251) were processed (IDs in Supplementary Table 9), the remaining sets were not evaluated due to the low number of accessions clustered with the exception of Gb to Gb (row by column) in Cluster 3.3, which reports 98% of Gb accessions clustered together and its results are considered highly similar to the results previously shown in Figures 4, 6 and reported in https://usda-ars-gbru.github.io/categorical_analysis_cotton/. Generally, the results were not greatly different than the previous bivariate association analysis results, so will not be discussed further, detailed results are available in Supplementary Figure 7 and Supplementary Table 10.

### Multiple Correspondence Analysis to Extract Information Content of Descriptors

**FIGURE 9:**
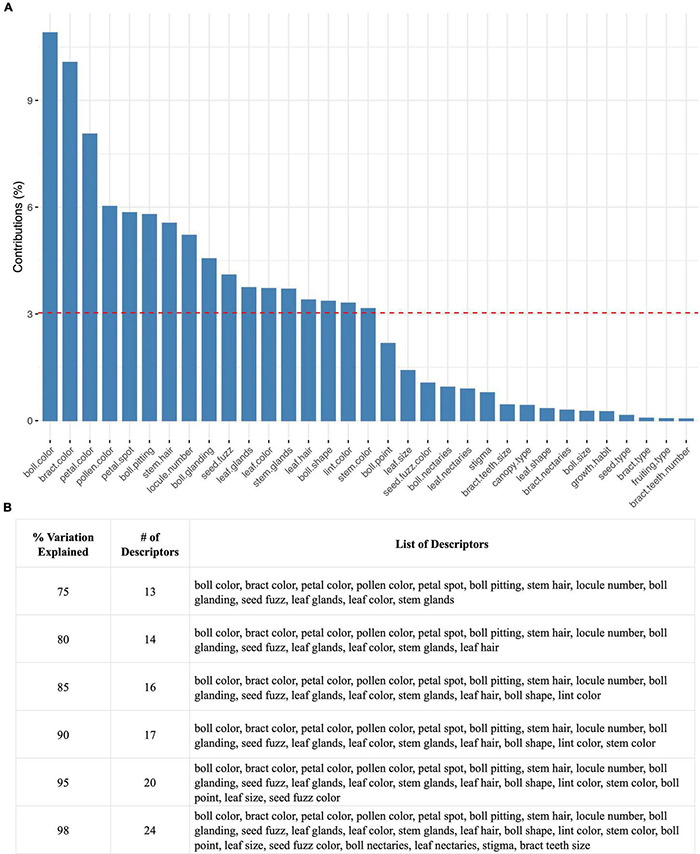
Descriptor contribution across the SA, TEX, and Gb groups. **(A)** Overview of the percent variation contributed to the overall dataset per categorical descriptor. **(B)** Group of categorical descriptors required to capture 75, 80, 85, 90, 95, and 98% of the overall dataset.

The calculation of how much each descriptor contributes to the total variation captured by a given Principal component is reported in Figure 9 for the whole three group set. (Values and corresponding charts are provided for each of the three groups separately, Supplementary Table 11 and Supplementary Figure 8. The cloud of descriptor correlations is reported in Supplementary Figure 9). The top contributing descriptors are boll color, bract color, and petal color (Figure 9A). The red dashed line indicates the expected average contribution (100% contribution divided by the total number of variables available in the dataset). Overall, 14, 17, and 24 descriptors can provide 80, 90, and 98 percent of the total variation captured in the overall dataset (Figure 9B).

## Discussion

Phenotypic descriptors are normally used to catalog plants in the United States GRIN-global germplasm system and for plant registrations. The standardized system that was developed for evaluating phenotypic information for cotton accessions in the NCGC allows for tracking diversity in a unique way while giving stability and evaluation robustness to the germplasm collection data (see Text Footnote 2). The standardized descriptors reflect phenotypic differences between cultivated materials and accessions of other origins that have been deposited in the collection.

### Categorical Descriptors Adequately Capture Diversity Between Pima and Upland Cotton

In total, 22% of the total unique accessions in the NCGC were evaluated in this study. The set of accessions evaluated were selected under the criteria that all of the standardized categorical descriptors had been collected for each one because missing information would have reduced statistical power and increased the chances of biased estimates and invalid conclusions. Even though only part of the NCGC cotton collection was analyzed, the high-quality data in the filtered dataset allowed us to draw overall conclusions about phenotypic variation within the SA, TEX, and Gb groups.

The unsupervised two cluster analysis for 33 categorical descriptors adequately separated more than 97% of the Pima and Upland accessions as originally described in the NCGC (*k* = 2) (Table 2). The remaining accessions that were clustered in the opposite group had a combination of descriptors not typical of their previously assigned species in NCGC, which could be due to handling or labeling errors in such a large germplasm collection or to unusual combinations of phenotypes, potentially arising through interspecific crosses or introgression. In certain environments or plant developmental stages, observations of potentially variable traits in hybrids could result in an error or ambiguity in species classification. Overall, this method of clustering accessions based on standardized descriptors can point to accessions within large germplasm banks that need more detailed analysis in order to identify unique and potentially useful genetic combinations and/or to improve the accuracy of the collection records.

The unsupervised three cluster analysis also clearly separated the Gb group, while showing more nuanced outcomes for the groups dominated by Gh accessions: 7.7% of the SA accessions were assigned to the TEX cluster and 33% of the TEX accessions were assigned to the SA cluster. The SA group is referred to as a germplasm breeding reference and contains many cultivars, whereas the TEX group is described as landraces or other tropical materials (Percy et al., 2014). These results are consistent with more extensive breeding to generate Gh cultivars. Early Gh domestication started with ancestors of the landraces that are commonly represented in the TEX group. In addition, more advanced germplasm from Mexico and Central America became an important resource in United States cotton selection and breeding programs beginning in the early 1800s (Moore, 1956; Wendel et al., 2010). Many of these introductions into United States cotton breeding were likely phenotypically quite close to modern Gh cultivars, except for environmentally responsive traits like photoperiodicity that would have been selected against in northerly regions and that were not included in our analysis. Logically, new combinations of phenotypes developed as cotton selection and breeding proceeded over time. Bivariate association analysis may have revealed differences in the composite plant traits between more primitive and advanced accessions as viewed from the cotton breeding perspective (Table 3). Genetic information can potentially augment the use of categorial descriptors as described here in further classifying the TEX accessions.

### Breeding Has Significantly Impacted the Way Phenotypes Are Associated

Statistical analysis of categorical descriptors collected by the NCGC shows that the breeding process in producing cotton cultivars (SA) has been modifying and reducing the number of significant ‘*descriptor_1:descriptor_2’* associations compared to the Upland cotton landrace accessions (TEX) and the Pima accessions (Gb). In contrast, most qualitative descriptors have some statistical association with others in the two predominantly Gh groups (SA and TEX) and the Gb group evaluated here (Figure 6). Interestingly, the low number of associations in SA is consistent with more extensive breakage of linkages between phenotypes that were originally present in *Gossypium* as compared to TEX which has seen less human manipulation. This is likely due to cotton breeders focusing on many different individual plant traits over time in response to biotic or abiotic stresses. In addition to focusing on specific traits, public breeders have introduced crosses focused on broadening the genetic base of Upland cotton. For example, they have begun evaluating accessions across different environments and looking to exotic or unusual germplasm present in the NCGC for new sources of diversity.

Across the three-group comparison, there were only three shared associations: ‘*bract nectaries:boll nectaries*’, ‘*leaf hair:stem hair*’, and ‘*lint color:seed fuzz color*’ (Figure 6A). Therefore, in these particular trait combinations, the association of particular phenotypes across the paired descriptors have not been broken within the accessions analyzed, including Gh and Gb accessions arising through modern breeding. In the case of the ‘*bract nectaries:boll nectaries’* association, the comparison between groups summarized in the results section suggests that it is uncommon for there to be a difference in presence or absence between boll and bract nectaries in the same accession. This may point to commonalities in the genetic control of nectary formation in both tissues. Despite these persistent pairings, the traits showed a wide variation of states within the range. Such observations are usually explained by polygenic effects (Waghmare et al., 2005; Hou et al., 2013; Hu et al., 2020). Also, we observed significant bivariate associations between descriptors with no obvious relationships, which could be due to pleiotropic effects when a gene product interacts with multiple others. The currently reported results lead to many future pathways of research to explore the genetic basis of the reported associations, such as the ‘*canopy type:fruiting type*’ significant association case, which is only reported in SA and Gb. Moreover, the canopy type trait reports multiple bivariate associations with other traits in TEX and GB, and independently for TEX, and GB.

The information that we investigated about the diversity of fiber and fuzz color, leaf and stem hairs, nectaries, and boll glands provides additional evidence that the germplasm material serves as a valuable resource in breeding materials for particular traits of interest which are associated with disease resistance, quality, growth habits, and ornamental interests, among others. For example, the data reported about the strong statistical association of presence/absence of nectaries, glands, seed fuzz, and plant hairs allows an interested breeder to identify the accessions with particular physiological conditions showing atypical distribution frequencies to independently explore the biological mechanisms involved in the anomalies of its physiological conditions, such as in the case of ‘*bract nectaries:boll nectaries’* there are 7 SA lines having present nectaries on bracts but absent on bolls (SA-1009, SA-1034, SA-2242, SA-2861, SA-2870, SA-2925, and SA-3611) and 3 SA lines with the opposite (SA-2946, SA-3570, and SA-3585). These particular trait associations could be targeted specifically for breakdown among elite materials as it potentially indicates there may either be very homogeneous genetic loci shared or in linkage disequilibrium among all the materials which limits potential diversity among other traits of interest shared in those genetic regions or the traits are controlled by some or all the same causal variants. Both factors play a role in this categorical study but exploring those conditions including genetic data could expand the understanding of the mechanisms associated with the traits that breeders could exploit to determine genotype-phenotype patterns. Currently the genomic data is not available for the NCGC but represents a potential future avenue of this research.

We were interested if we could better understand the historical contribution of the materials in this study to cotton breeding and research, which may have targeted use of these materials for certain desired traits as outlined above. The NCGC has tracked the number of total seed requests for each line since 2007 (Supplementary Tables 2–4), which should correlate with the utilization of a line in practice. The SA collection has seed request numbers from 0 to 47, averaging 4.5 ± 5.5 requests per line. The TEX collection has seed request numbers from 0 to 20, averaging 5.8 ± 3.2 requests per line. The GB collection has seed request numbers from 0 to 57, averaging 4.3 ± 5.9 requests per line. There were a few major standouts in the SA and GB collections as the most requested lines. In SA, the most requested line is Coker 310 (47 requests); which is an important line from which Coker 312 was selected from, as the most regenerable line of cotton (Trolinder and Goodin, 1987; Bowman et al., 2006). The next most requested lines both have the green lint phenotype, Arkansas Green Lint (42 requests) and Intense Red Green Lint (36 requests), which reflects the interest in cotton that does not require dyeing (Vreeland, 1999). In Gb, the most requested lines are Pima S-6 (57 requests) and Pima S-7 (55 requests), they both have long fiber, good yield and are earlier maturing than most *G. barbadense* lines (Feaster and Turcotte, 1984; Turcotte et al., 1992). The lines have also been studied for their reaction to important diseases such as verticillium wilt and fusarium wilt (Bolek et al., 2005; Wang and Roberts, 2007; Zhu et al., 2021). The third most requested line is Bleak Hall Sea Island (37 requests), an important genetic contributor to the USDA-ARS Pee Dee Breeding Program focused on fiber quality (Campbell et al., 2011). In a field trial of 48 Pima lines, it had the longest fiber length at 37.8 mm (Holladay et al., 2021). The presence of a registration in the Plant Variety Protection (PVP) system often indicates the importance of a line. Of lines studied here, there are only 2 lines that are ex-PVP materials (lines for which a PVP was filed and ex indicating they have passed the time of legal protection), both in the SA collection, Stoneville 907 (*PVP - Stoneville 907, n.d.*) and DP 5409 (*PVP - DP5409, n.d.*). Therefore, it is likely the seed request data provides more data on the importance of the study materials to historical cotton breeding and research.

### Resistance-Associated Phenotypes Show Different Patterns of Relationship Among Stoneville Accessions, Texas Accessions, and *Gossypium barbadense*

Gossypol glands play an important role in insect resistance because gossypol is often toxic. The glands are considered direct resistance traits because the plant invests directly in its own defense (Rudgers et al., 2004). In cultivated cotton, the presence and density of glands, which may be found on leaves, stems, and/or bolls, are negatively correlated with the abundance, performance, and/or damage caused by several herbivores (Matthews, 1989; Summy and King, 1992). Results (Supplementary Figure 6) showed that both SA and TEX have significant bivariate associations for ‘*leaf glands:boll glands’* and ‘*leaf glands:stem glands’*, while ‘*boll glands:stem glands’* are only significantly associated in the TEX group (Figure 6). Most of the accessions in all three groups had at least medium glanding on all three organs (Figure 3), which is consistent with a positive impact of glands on defense against insects. Most SA accessions had medium glanding on leaves, stems, and bolls, and rarer cases had glandless leaves and bolls. In contrast, the TEX group contained accessions with glandless bolls accompanied by medium and heavy leaf glands. The majority of TEX accessions had medium glanding in bolls and heavy glanding in leaves and stems. In the Gb group, 98% of the accessions were rated as ‘heavy’ for the glanding on all three organs (Figure 3). Therefore, more extensive breeding in the SA group has led to lesser glanding overall as compared to TEX or Gb. These findings are reasonable from the perspectives of adaptation and evolution because glands provide the plant with natural protection from insects. Thus, losing the glanding trait would be detrimental to overall plant fitness and make it difficult for a breeder to impact glanding. This study is consistent with previous efforts showing the difficulty of breeding for reduced glanding, potentially indicating alternative breeding methods should be applied where gland modification is the goal (Janga et al., 2019; Gao et al., 2020).

Extrafloral structures such as nectaries reflect indirect resistance mechanisms because the plants invest in interactions with other species (Rudgers et al., 2004). The ‘*bract nectaries:boll nectaries’* association was significant in all three groups analyzed. The different biological backgrounds of each class and the states observed for descriptors showed differences and similarities in its range trait relationship. The present and reduced states are the most common conditions across the three groups, with absence of bract and boll nectaries only rarely observed among the accessions analyzed. The presence of nectaries could be considered an advantage or disadvantage depending on the natural conditions of the individual in the wild or its use for breeding purposes.

These descriptor traits may be more valuable in ranges where cotton production and specific environmental factors ranges overlap, such as native insect ranges. Assessment of accession geographic collection information, or georeferencing, has led to valuable insights particularly in botanical studies (Swenson et al., 2012; Choudhary et al., 2022). Crop species have a particular difficulty in utilizing geographic data as many accessions were obtained outside of collection expeditions thus contain uninformative or inaccurate geographic data, extensive data filtering would be required to even potentially utilize that data (Feeley and Silman, 2010), but may be worth investigating in the future to potentially add value to the germplasm collection (Volk and Richards, 2011).

### Leveraging Germplasm Collection Systems

This analysis expands the use of categorical descriptors normally used for cataloging cotton accessions or germplasm registration. We show that computational and statistical analysis can allow categorical data to be used for illustration and exploration of diversity, trait associations, and similarity in the cotton germplasm collection. The robustness of the analysis is based on the standardized systems developed by the germplasm curators to track multiple phenotypic traits of cotton accessions planted annually in different environments. This research is only based on categorical data and helps to understand the heterogeneity of the cotton accessions present in the collection. This information can be used by the breeding community to integrate new material with desirable traits or unique trait combinations into their breeding programs. While this analysis focused on categorical data as this is the prevalent information available on large numbers of individuals in germplasm collections, a similar strategy can be applied to quantitative data and many of the tools used here are suitable for quantitative data (Lê et al., 2008; Husson et al., 2010; Akay and Yüksel, 2017). As larger quantitative data sets are available for germplasm collections, it will also be possible to combine qualitative and quantitative data for analysis.

We analyzed accessions representing 2 of the over 50 *Gossypium* species represented in the NCGC. The NCGC is only one of 44 collections with over 500,000 unique accessions representing over 10,000 species in the GRIN-global germplasm system [(dataset) USDA Agricultural Research Service, 2015], with different curators all collecting similar categorical descriptor data on the different crop-specific germplasm collections. While determining the specific number of categorical traits necessary to be informative for a collection will be collection specific, the analytical methods and refined insights about the collection demonstrated in this study could be extended to other crops or organisms present in the GRIN-global system. We would suggest a researcher to systematically collect the largest possible set of descriptor traits on a smaller diversity panel or core set of accessions, then use multiple correspondence analysis as outlined here to understand the most informative set of descriptors to collect on the larger collection. A better understanding of germplasm collections will allow for more effective use of these resources and help to safeguard the genetic diversity of agriculturally important plants, which is essential for protecting agriculture in the future (FAO, 2010; Byrne et al., 2018).

## Data Availability Statement

The datasets presented in this study can be found in online repositories. The names of the repository/repositories and accession number(s) can be found below: GitHub (https://usda-ars-gbru.github.io/categorical_analysis_cotton/), GRIN (https://npgsweb.ars-grin.gov/gringlobal/crop?id=547), and Cottongen (https://www.cottongen.org/).

## Author Contributions

DR-M, AMH-K, JAS, DCJ, and JF conceived the project. JAS, LLH, JL, RGP, and JF managed field locations and data collection, and managed the germplasm collection. DR-M analyzed the data. DR-M, AMH-K, JAS, and CHH synthesized and interpreted the results and wrote the manuscript. All authors edited and approved the manuscript.

## Author Disclaimer

Mention of trade names or commercial products in this publication is solely for the purpose of providing specific information and does not imply recommendation or endorsement by the U.S. Department of Agriculture.

## Conflict of Interest

The authors declare that the research was conducted in the absence of any commercial or financial relationships that could be construed as a potential conflict of interest.

## Publisher’s Note

All claims expressed in this article are solely those of the authors and do not necessarily represent those of their affiliated organizations, or those of the publisher, the editors and the reviewers. Any product that may be evaluated in this article, or claim that may be made by its manufacturer, is not guaranteed or endorsed by the publisher.

## Footnotes

1
https://npgsweb.ars-grin.gov/gringlobal/crop?id=547

2
https://www.cottongen.org/data/trait/NCGC_rating_scale

3
https://www.grin-global.org

4
https://usda-ars-gbru.github.io/categorical_analysis_cotton/

5
https://www.jmp.com/en_us/statistics-knowledge-portal/exploratory-data-analysis/mosaic-plot.html

## Supplementary Material

Supplementary Figure 1 Categorical state distributions for the 33 traits evaluated.

Supplementary Figure 2 Heatmap of the bivariate descriptor associations for TEX.

Supplementary Figure 3 Heatmap of the bivariate descriptor associations for Gb.

Supplementary Figure 4 Leaf hair – stem hair analysis.

Supplementary Figure 5 Lint color – seed fuzz color analysis.

Supplementary Figure 6 Boll glanding – leaf glanding – stem glands analysis.

Supplementary Figure 7 Heatmaps of SA-251, TEX-308, and TEX-156 sets.

Supplementary Figure 8 Multiple correspondence analysis for SA, TEX, and Gb.

Supplementary Figure 9 Cloud of descriptor correlations.

Supplementary Table 1 Standardized descriptors and rating reported by USDA-ARS College Station.

Supplementary Table 2 SA_mod.xlsx – input dataset for bivariate analysis.

Supplementary Table 3 TEX_mod.xlsx – input dataset for bivariate analysis.

Supplementary Table 4 gb_mod_PAG.xlsx – input dataset for bivariate analysis.

Supplementary Table 5 Categorical descriptors encoded for bivariate and clustering analysis.

Supplementary Table 6 Modified states in categorical analysis for statistical purposes.

Supplementary Table 7 Integrated dataset of SA, TEX, and Gb accessions for unsupervised clustering.

Supplementary Table 8 Two cluster results and IDs identified.

Supplementary Table 9 Three cluster results and IDs identified.

Supplementary Table 10 All FDR *p*-values for the SA, TEX, Gb, SA-251, TEX-156, and TEX-308 sets.

Supplementary Table 11 Values and corresponding charts are provided for SA, TEX, and Gb.

Supplementary Data 1 Updating and expanding definitions and rating system.

Supplementary Data 2 “One_hot-ordinal.py” script to transform data to apply the clustering analysis.

Supplementary Data 3 “MCA_ind_corr_contr_sa_tex_gb.R” script to calculate MCA and plot results.
