# Supplementary figures and images for: Leveraging National Germplasm Collections to Determine Significantly Associated Categorical Traits in Crops: Upland and Pima Cotton as a Case Study
Source: Front Plant Sci. 2022 Apr 26;13:837038. doi: 10.3389/fpls.2022.837038 (PMC9087864; doi:10.3389/fpls.2022.837038)

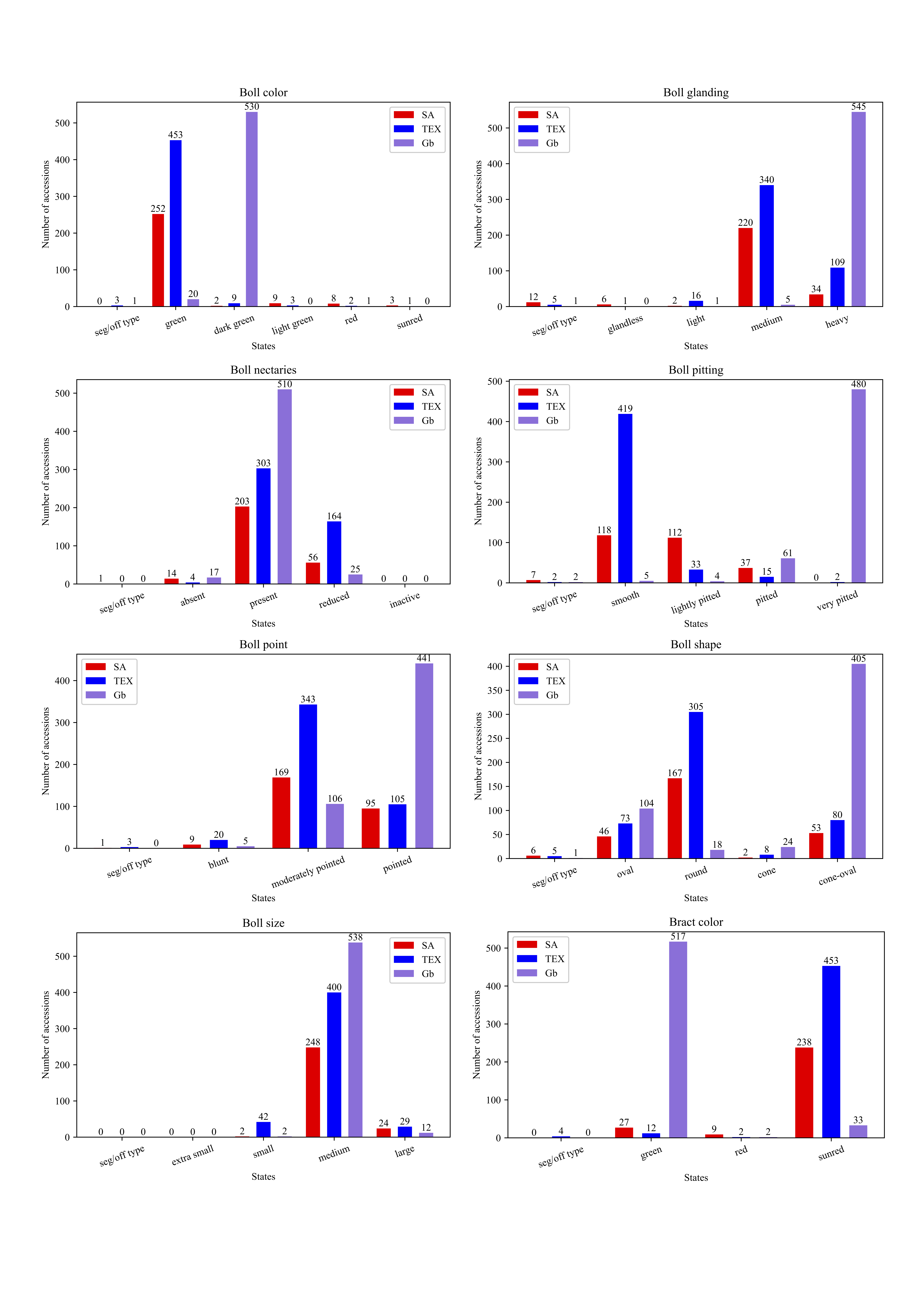

Supplement: Supplementary Figure 1 — Categorical state distributions for the 33 traits evaluated. [file Image_1.JPEG]

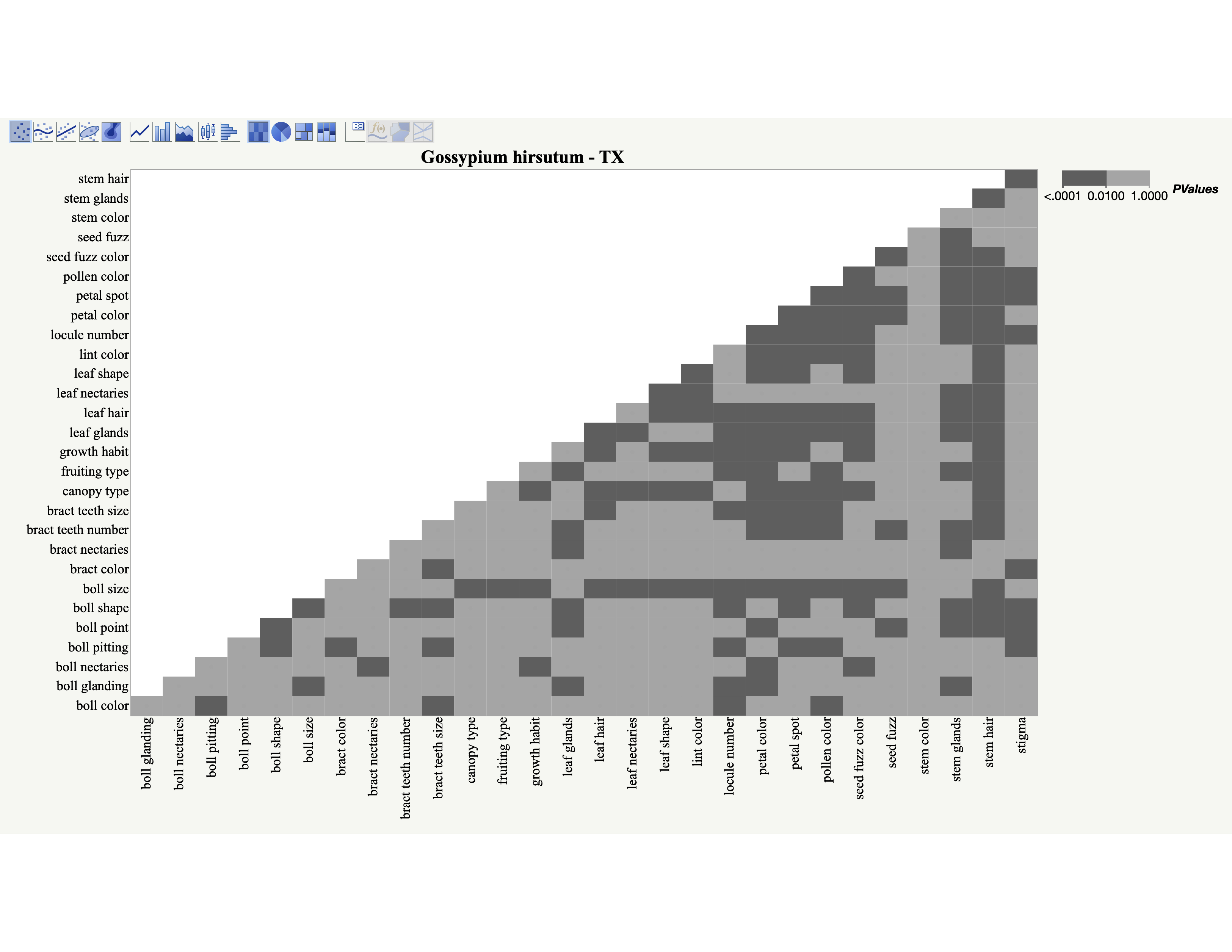

Supplement: Supplementary Figure 2 — Heatmap of the bivariate descriptor associations for TEX. [file Image_2.JPEG]

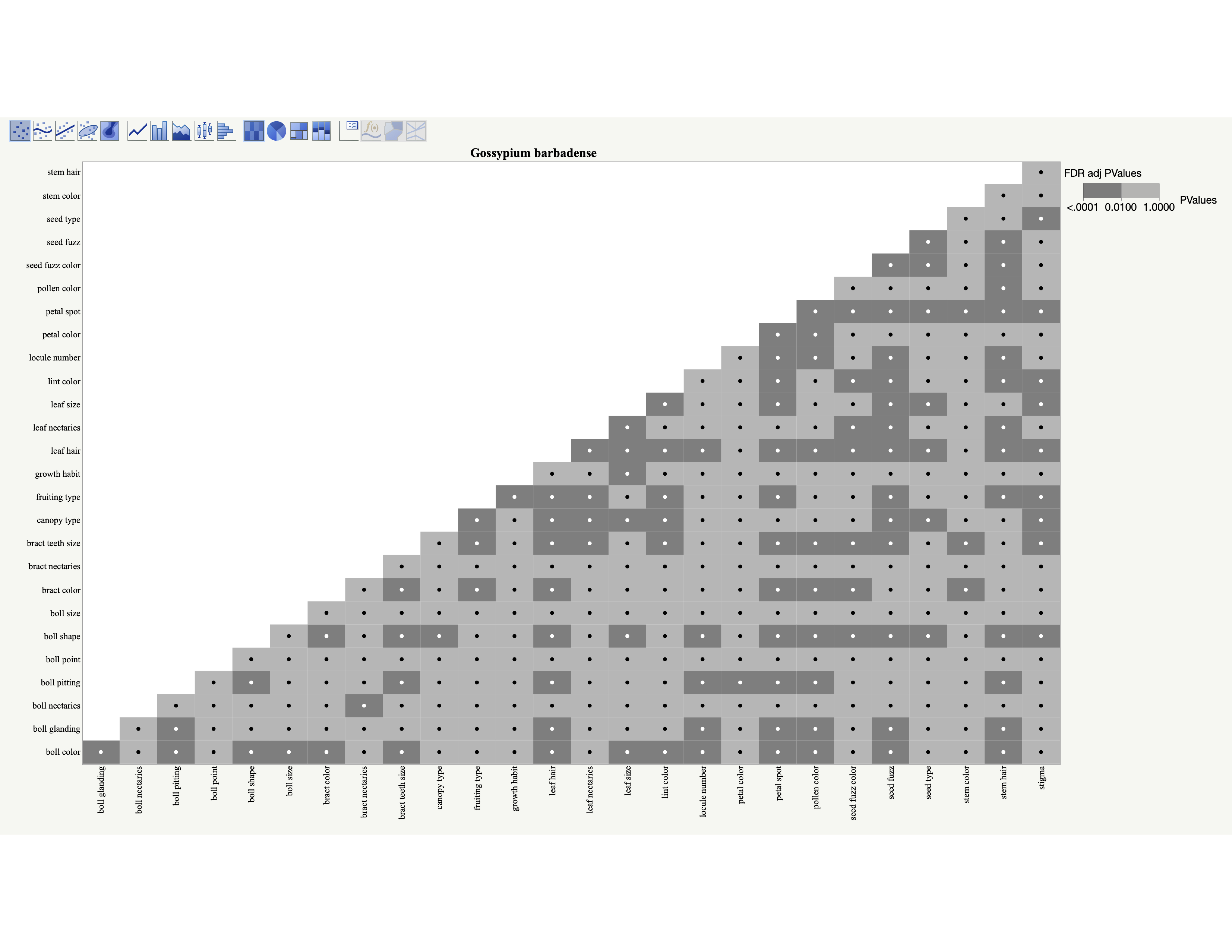

Supplement: Supplementary Figure 3 — Heatmap of the bivariate descriptor associations for Gb. [file Image_3.JPEG]

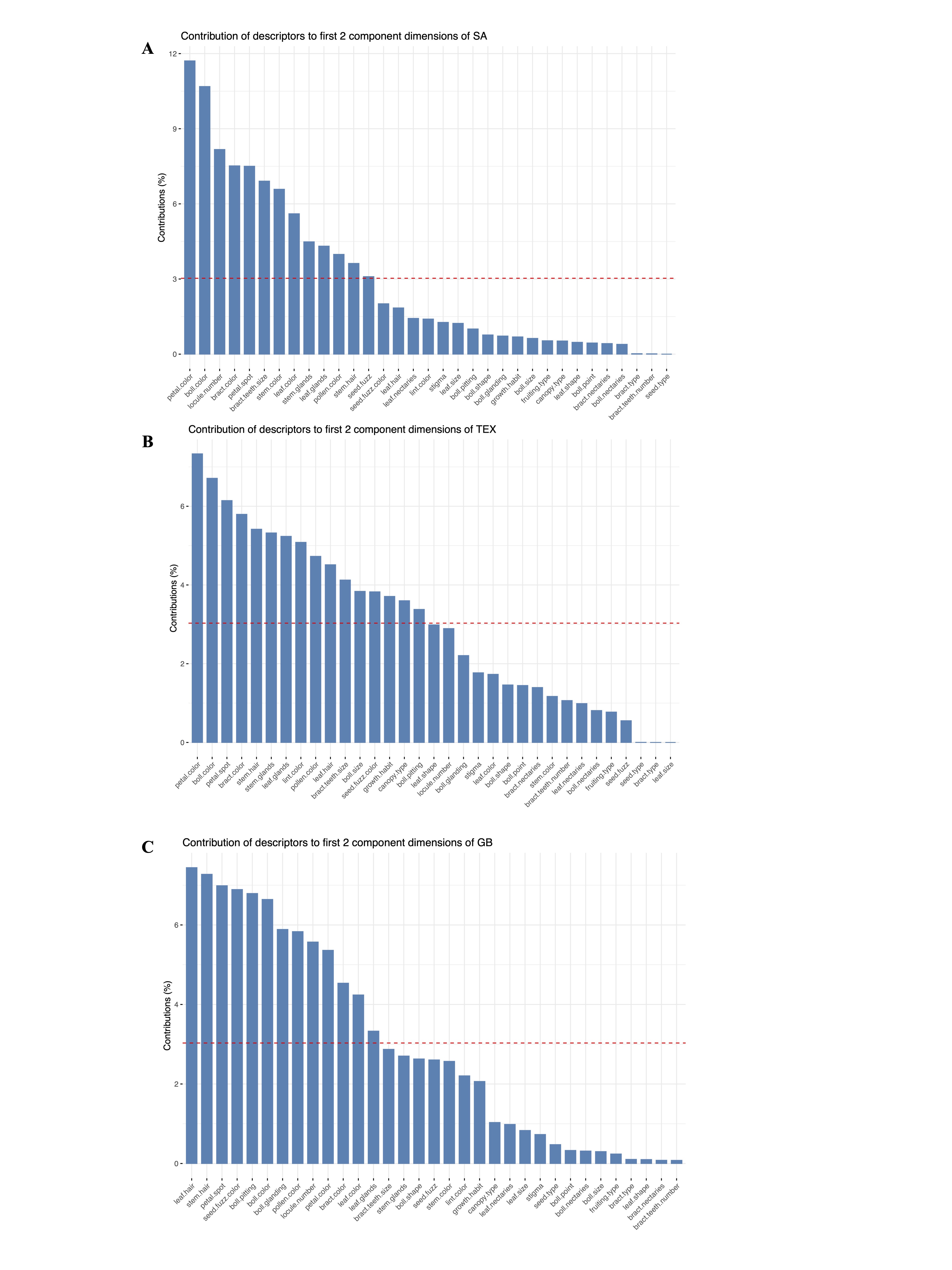

Supplement: Supplementary Figure 8 — Multiple correspondence analysis for SA, TEX, and Gb. [file Image_8.JPEG]

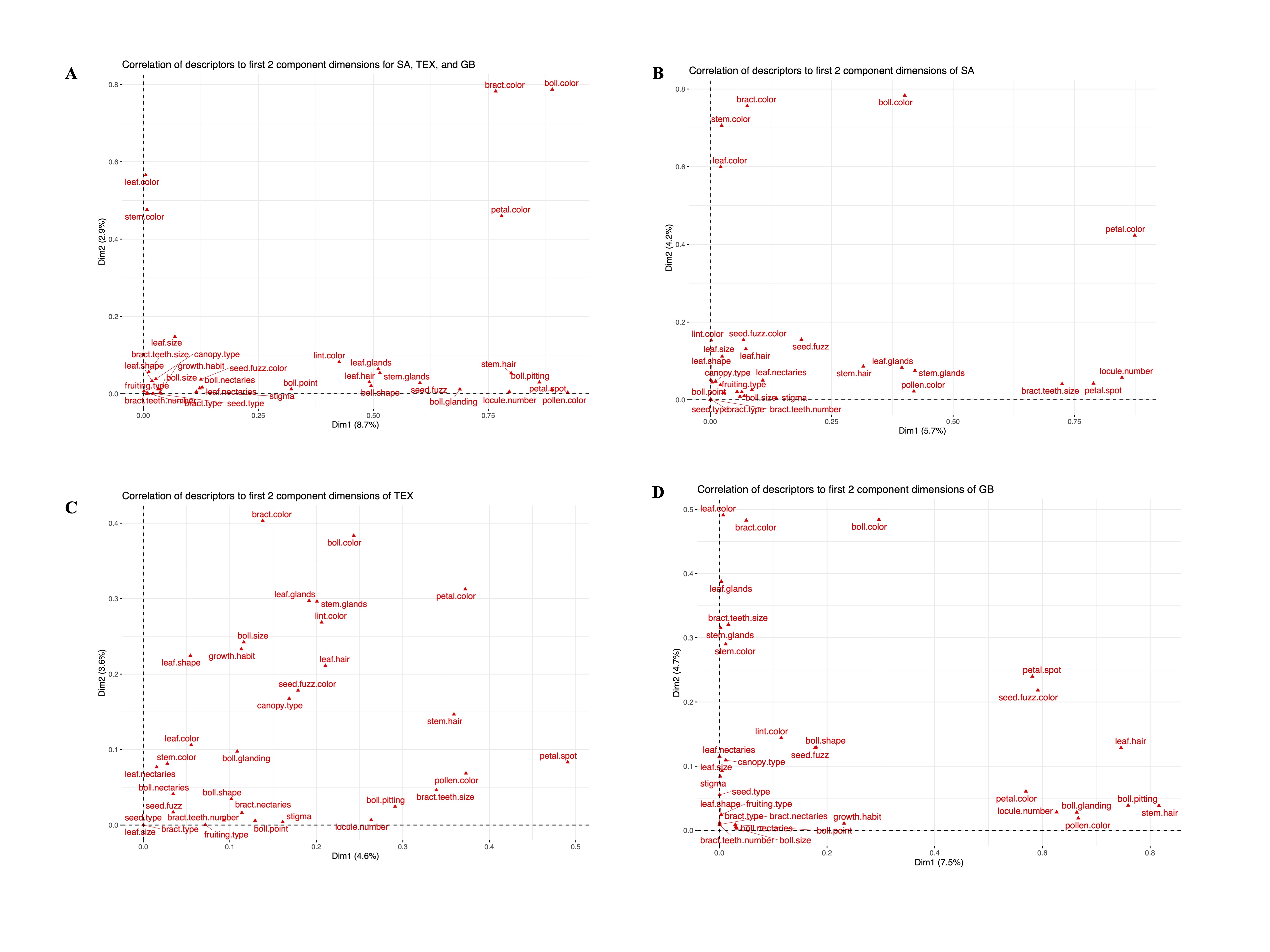

Supplement: Supplementary Figure 9 — Cloud of descriptor correlations. [file Image_9.JPEG]
