# Supplementary material for: Leveraging National Germplasm Collections to Determine Significantly Associated Categorical Traits in Crops: Upland and Pima Cotton as a Case Study
Source: Front Plant Sci. 2022 Apr 26;13:837038. doi: 10.3389/fpls.2022.837038 (PMC9087864; doi:10.3389/fpls.2022.837038)
Supplement: Supplementary Table 1 — Standardized descriptors and rating reported by USDA-ARS College Station. [file Data_Sheet_4.PDF]

Standardized Descriptors and Rating Scale used for the National Cotton Germplasm Collection

Data provided by **USDA-ARS College Station, TX, USA,** Nov. 2014 and updated May 2017.
*font **green** indicates the descriptor has panel image available (to view the panel image, click on the descriptor's name).

Descriptor	Description Code or Rating Scale									
	0	1	2	3	4	5	6	7	8	9
Boll Color*		green	dark green	light green	red	sun red				seg/off type
Boll Glanding	glandless	medium	light	heavy						seg/off type
Boll Nectaries	absent	present	reduced	inactive						seg/off type
Boll Pitting		smooth	light pitted	pitted	very pitted					seg/off type
Boll Point		Moderately pointed	pointed	blunt						seg/off type
Boll Shape		Oval	round	cone	cone-oval					seg/off type
Boll Size		medium	large	small	extra small					seg/off type
Bract Color		green	red	sun red						seg/off type
Bract Teeth Number		medium	many	few						seg/off type
Bract Teeth Size	none	medium	small	large						seg/off type
Bract Type		normal	frego	flared	recurved					seg/off type
Fruiting Type		normal	cluster							seg/off type
Growth Habit		normal	spreading	prostrate	pyramid	stovepipe				seg/off type
Leaf Canopy		typical	open	dense	compact					seg/off type
Leaf Color		green	red	dark red						seg/off type
Leaf Glands	glandless	medium	light	heavy						seg/off type
Leaf Hairs		none	few	moderate	hairy	very hairy	pilose			seg/off type
Leaf Nectaries	absent	present one of following: one, main vein(1-1), two(1-2), three(1-3), four(1-4), five(1-5)					reduced (2)			seg/off type
Leaf Shape		normal	okra	sub okra	super okra	laciniate	ovate	cordate		seg/off type
Leaf Size		medium	small	large	extra small					seg/off type
Lint Color		white	cream	brown	green	tan	rust	off white		seg/off type
Locule #		one	two	three	four	five	> five			seg/off type
Maturity		not flowering	flowering	< 1/2 open	> 1/2 open	complete				seg/off type
Petal Color		cream	yellow	light yellow	red	white	light blue	golden	pink	seg/off type
Petal Spot	none	light	medium	heavy						seg/off type
Photoperiodic Rating	no squares	squares	flowers	bolls	open bolls					seg/off type
Plant height (m)	actual measurement									
Pollen Color		yellow	cream	--	dark yellow	orange				seg/off type
Productivity	none	< 12/plant	13-24/plant	25-36/plant	37-48/plant	>48/plant				seg/off type
Seed Fuzz	none	medium	high	sparse	tufted					seg/off type
Seed Fuzz Color	lintless	white	cream	brown	green	tan	rust	off white		seg/off type
Seed Type		free	semi kidney	kidney						seg/off type
Stem Color		green	sun red	red						seg/off type
Stem Glands	glandless	medium	light	heavy						seg/off type
Stem Hair		none	few	moderate	hairy	very hairy	pilose			seg/off type
Stigma		normal	protruding	extreme protruding	short buried					seg/off type
