# Supplementary material for: Leveraging National Germplasm Collections to Determine Significantly Associated Categorical Traits in Crops: Upland and Pima Cotton as a Case Study
Source: Front Plant Sci. 2022 Apr 26;13:837038. doi: 10.3389/fpls.2022.837038 (PMC9087864; doi:10.3389/fpls.2022.837038)
Supplement: Supplementary Table 6 — Modified states in categorical analysis for statistical purposes. [file Data_Sheet_5.PDF]

Table S6. Modifications of the levels in categories/traits for bivariate associations analysis.

Category	***SA***	***TEX***	***Gb***
Growth Habit^	“Pyramid” and “Stovepipe”:removed	Process without modification	“Spreading”:removed
Canopy Type^	“Dense”:removed	Process without modification	“Compact”:removed
Leaf Hairs*	“Pilose”:removed	Process without modification	“Hairy” merged:“Hairy” and “Very Hairy”
Leaf color^	“dark_red”:removed	Not evaluated	Not evaluated
Leaf Shape^	“~okra” merge: “okra”, “subokra”, and “superokra”	“subokra”:removed	Not evaluated
Stem Color^	Process without modification	“green”:removed	“Green”:removed
Stem Glands*	“glandless”:removed	“Glandless” “light”:removed	Not evaluated
Stem Hair*	“veryhairy”:removed	Process without modification	“Hairy” merged: “Hairy” and “Very Hairy”. “Moderated”: removed
Leaf Size*	Not evaluated	Not evaluated	Process without modification
Leaf Glands*	Process without modification	“Glandless” “light”:removed	Not evaluated
Leaf Nectaries* eval:02/13/2020 - replace “inactive” for reduce	“Absent” ’’reduced’:removed Low frequency	’’Reduced””four”:removed Absent - not present	“Absent”: removed Reduced - not present
Bract Nectaries^ eval:02/13/2020 - replace “inactive” for reduce	Process without modification Not identified	“Absent”:removed Not identified	Process without modification Not identified
Boll nectaries^	Process without modification	“absent”:removed	Process without modification
Petal Color^	Process without modification	“red”:removed	“Cream”:removed
Pollen Color^	“Yellow” merged: “yellow” and “darkyellow”	Process without modification	“Orange”:removed

Petal Spots*	“Spots” merged: “light”, “medium”, and “heavy”	“heavy”:removed	“Light”: removed
Stigma*	Process without modification	Process without modification	“Extreme protruding”: removed
Lint Color^	“Color” merged: ”brown” “green”, and “tan”	“Cream” “rust” “offwhite”:removed	“Tan”, “rust”, and “off white”: removed
Locule Number*	“Three”: removed	Process without modification	“Five”: removed
Seed Fuzz*	“none”:removed	Process without modification	Process without modification
Seed Fuzz Color^	“lintless”:removed	“rust”:removed	“White” and “Rust”: removed
Seed Type^	Not evaluated	Not evaluated	Process without modification
Bract Type^	Process without modification	Not evaluated	Not evaluated
Bract Teeth Size*	Not evaluated	“small”:removed	Process without modification
Bract Teeth Number*	Not evaluated	“many”:removed	Not evaluated
Bract Color^	Process without modification	“red”:removed	“Red”:removed
Boll Shape^	“cone”:removed	Process without modification	Process without modification
Boll Point*	Process without modification	Process without modification	Process without modification
Boll Size*	“small”:removed	Process without modification	“Small”: removed
Boll Color^	“darkgreen” “sunred”:removed	“Lightgreen” ”red” “sunred”:removed	“Red”: removed
Boll Glanding*	“light”:removed	“glandless”:removed	“Light”: removed
Boll Pitting*	Process without modification	“verypitted”:removed	“Lightly pitted”: removed
Fruiting Type^	Process without modification	“Shortbranch”:removed only 1 reported - change stovepipe	Process without modification Not identified

mod:02/13/2020 - replace “inactive” for reduce			

^nominal, *ordinal. Not evaluated: categories with information in one range only. Removed: state with less than 5 observations.
