# Supplementary material for: Leveraging National Germplasm Collections to Determine Significantly Associated Categorical Traits in Crops: Upland and Pima Cotton as a Case Study
Source: Front Plant Sci. 2022 Apr 26;13:837038. doi: 10.3389/fpls.2022.837038 (PMC9087864; doi:10.3389/fpls.2022.837038)
Supplement: Supplementary Data 1 — Updating and expanding definitions and rating system. [file Data_Sheet_1.PDF]

Data S1: COTTON Descriptors

Descriptors and Other Fields
(CTRL+click to jump to page)

MATURITY, numeric	3
PHOTOPERIODIC RATING, numeric	3
GROWTH HABIT, numeric	3
CANOPY/TYPE, numeric	4
LEAF HAIR, numeric	5
LEAF COLOR, numeric	5
LEAF SHAPE, numeric	5
STEM COLOR, numeric	6
STEM GLANDS, numeric	6
STEM HAIR, numeric	6
LEAF SIZE, numeric	7
LEAF GLANDS, numeric	7
LEAF NECTARIES, alphanumeric	8
BRACT NECTARIES, numeric	8
BOLL NECTARIES, numeric	8
PETAL COLOR, numeric	9
POLLEN COLOR, numeric	9
PETAL SPOT, numeric	9
STIGMA, numeric	10
LINT COLOR, numeric	10
LOCULE NUMBER, numeric	10
SEED FUZZ, numeric	11
SEED FUZZ COLOR, numeric	11
SEED TYPE, numeric	11
BRACT TYPE, numeric	12
BRACT TEETH SIZE, numeric	12
BRACT TEETH NUMBER, numeric	12
BRACT COLOR, numeric	12
BOLL SHAPE, numeric	13
BOLL POINT, numeric	13
BOLL SIZE, numeric	13
BOLL COLOR, numeric	13
BOLL GLANDING, numeric	14
BOLL PITTING, numeric	14

FRUITING TYPE, numeric 14

MATURITY, numeric

The stage of maturation of the plot at the time of observation. It helps to describe agronomic performance of the accession and is most useful if also scored on a set of cultivars standard to all planting plans.
not flowering (1) = flowers have not formed yet
flowering (2) = flowers have formed, but bolls are not yet forming
less than 1/2 open (3) = bolls have already formed, but most are unopened
more than 1/2 open (4) = most bolls that have formed are opening
complete (5) = all or nearly all the bolls that have formed are open or are opening (cracking)
seg/off type (9), rating to indicate that the plot is segregating for this trait and a single score is not an accurate description of the plot. Alternatively the rating is off the standard scale and best described as off type. Either is applicable to all the descriptors and best categorized this way and with same value (9). Comments may be added to clarify the multiple observations of this trait.

PHOTOPERIODIC RATING, numeric

Indication of the flowering of the accession in relation to daylength. Temperate or semi tropical regions have summers suitable for cotton production but daylengths exceed 12 hours which can inhibit flowering of accessions from tropical regions. Scores are most meaningful when collected around 3 weeks post Summer Solstice and in relation to additional descriptions such as first flowering Days After Planting and interpreted in light of the number of weeks that flowering continued in the growing season, because the cessation of spurious, early flowering is also an indicator of photoperiodic response.
no squares (0) = no sign of flowering response in the plot
squares (1) = flower buds are formed in the plot
flowers (2) = flowers are opening, but bolls are not yet formed
bolls (3) = bolls are developing on the plants
open bolls (4) = bolls are already maturing on plants
seg/off type (9), rating to indicate that the plot is segregating for this trait and a single score is not an accurate description of the plot. Alternatively the rating is off the standard scale and best described as off type. Either is applicable to all the descriptors and best categorized this way and with same value (9). Comments may be added to clarify the multiple observations of this trait.

GROWTH HABIT, numeric

General classes of some characteristic patterns of stem growth of the plants. Physical growth patterns of the stem and branches can impact yield and ability to harvest an accession.
normal (1) = stem growth is typical of that seen for most cotton cultivars or for lack of a better descriptor for some wild species, it is the wild form
spreading (2) = stem and branch growth tends to be top heavy
prostrate (3) = stem and branch growth is close to the ground
pyramid (4) = stem growth is orderly, often with a single main stem, and takes precedence over branch growth. It is typical of accessions classified as landrace 'Palmeri' and some tree like species of *Gossypium*.
stovepipe (5) = branch growth is highly suppressed leading to single stemmed and very narrow plants. Short branch fruiting character is often seen.
seg/off type (9), rating to indicate that the plot is segregating for this trait and a single score is not an accurate description of the plot. Alternatively the rating is off the standard scale and best described as off type. Either is applicable to all the descriptors and best categorized this way and with same value (9). Comments may be added to clarify the multiple observations of this trait.

CANOPY TYPE, numeric

The overall appearance of foliage on the plants. For a cotton breeder it can reflect the final quality of cotton, because a high concentration of leaves close to the bolls result in more trash, insect debris and mold growth.

typical (1) = foliage cover is typical of that seen in most cotton cultivars or for lack of a better descriptor for some wild species, it is the wild form

open (2) = leaves are spaced further than that seen for most cotton cultivars. Long internodes and/or small or dissected (e.g. okra leaf shape) leaves contribute to the open canopy

dense (3) = close placement of leaves on the plant. Often applied to accessions further classified as landrace "Morrilli" where the branches are close together.

compact (4) = close placement of leaves and likely caused by genetic dwarfism and short internodes

seg/off type (9), rating to indicate that the plot is segregating for this trait and a single score is not an accurate description of the plot. Alternatively the rating is off the standard scale and best described as off type. Either is applicable to all the descriptors and best categorized this way and with same value (9). Comments may be added to clarify the multiple observations of this trait.

LEAF HAIR, numeric

The degree or relative density of hairs on the leaf surfaces. Focus is mainly on expanded leaves at the fourth node, or further, from the growing tip

none (1) = hairs appear to be absent from leaf surfaces

few (2) = leaf hairs are at a low frequency

moderate (3) = leaf hairs are at a regular and commonly seen density for most cotton cultivars

hairy (4) = leaf hair density is greater than that normally seen on most cotton cultivars. May lead to more adhering of leaf trash to harvested cotton, but in other regions such as in the African continent this character maybe essential for resistance to certain insects (e.g. Jassids)

very hairy (5) = highest density seen on leaf surfaces. For cotton cultivars it likely has been selected for key loci for overexpression

pilose (6) = leaf hairs are short and may lend a velvety feel to leaf surfaces. Relative density is harder to describe and may be re-examined with digital images and/or supplemented in the comments section. It is characteristic of the species *G. tomentosum*. Digital images are essential to confirm this trait in accessions which may have a variety of leaf hairs, differing in length and density.

seg/off type (9), rating to indicate that the plot is segregating for this trait and a single score is not an accurate description of the plot. Alternatively the rating is off the standard scale and best described as off type. Either is applicable to all the descriptors and best categorized this way and with same value (9). Comments may be added to clarify the multiple observations of this trait.

LEAF COLOR, numeric

The degree of red coloration to the leaf color. Red coloration has value as an ornamental, marker plant, or possibly increased resistance to certain pests.

green (1) = leaf is primarily green in color

red (2) = red coloration is regularly seen in the interveinal areas of mature leaves and ideally is not a byproduct of stress.

dark red (3) = red coloration permeates the entire leaf and is likely a result from selection for alleles to over express.

seg/off type (9), rating to indicate that the plot is segregating for this trait and a single score is not an accurate description of the plot. Alternatively the rating is off the standard scale and best described as off type. Either is applicable to all the descriptors and best categorized this way and with same value (9). Comments may be added to clarify the multiple observations of this trait.

LEAF SHAPE, numeric

The general shape of the mature, expanded leaves. It typically refers to the degree of dissection or lobing of the leaf.

normal (1) = leaf shape typically seen in cotton cultivars or for lack of a better descriptor in species, the wild type

okra (2) = leaf shape is more dissected than that normally seen. In cultivars it is controlled by known allele(s). Also applied to some species such as *G. arboreum*, *G. thurberi*, *G. trilobum*, and *G. bickii*

sub okra (3) = leaf shape is intermediate between normal and okra.

super okra (4) = leaf shape is highly dissected and lobing is sometimes absent and showing single leaflets

laciniate (5) = leaf shape includes spurs at the base of the leaf. Commonly seen in *G. arboreum*

ovate (6) = leaf shape is rounded or oval. Best seen in a number of Australian diploid species

cordate (7) = leaf shape is like an inverted heart shape, with a broad rounded base, tapering to a tip. Best seen in some diploid species, such as *G. aridum* and *G. longicalyx* for example

seg/off type (9), rating to indicate that the plot is segregating for this trait and a single score is not an accurate description of the plot. Alternatively the rating is off the standard scale and best described as off

type. Either is applicable to all the descriptors and best categorized this way and with same value (9). Comments may be added to clarify the multiple observations of this trait.

STEM COLOR, numeric

The degree of red coloration to the stems. Red coloration has value as an ornamental, marker plant or possibly to resist certain insects
green (1) = stem is primarily green in color
sun red (2) = red coloration is regularly seen on the side of the stem most exposed to the sun
dark red (3) = red coloration permeates the stem and likely resulting from selection for alleles to over express.
seg/off type (9), rating to indicate that the plot is segregating for this trait and a single score is not an accurate description of the plot. Alternatively the rating is off the standard scale and best described as off type. Either is applicable to all the descriptors and best categorized this way and with same value (9). Comments may be added to clarify the multiple observations of this trait.

STEM GLANDS, numeric

The relative density of gossypol glands on the stems. These are observed as the small dark dots on the stems. Gossypol glands impart resistance to some herbivores and pests, but complicate purification of oils and protein from seeds
glandless (0) = no observable dark glands on the stems. Most likely a result of intense selection against gossypol glands
medium (1) = typically seen density of gossypol glands for most cotton cultivars
light (2) = lower than normal density of gossypol glands. Likely was selected for alleles to underexpress.
heavy (3) = density of glands is greater than that normally seen in most cotton cultivars. Maybe a result of selection or is common for some species, such as *G. barbadense* and *G. arboreum*
seg/off type (9), rating to indicate that the plot is segregating for this trait and a single score is not an accurate description of the plot. Alternatively the rating is off the standard scale and best described as off type. Either is applicable to all the descriptors and best categorized this way and with same value (9). Comments may be added to clarify the multiple observations of this trait.

STEM HAIR, numeric

The relative density of hairs on the stems. Focus is mainly on region of the stem at the fourth node from the growing tip. Stem tip pictures are taken to show this and any variation that may occur such as loss of hairs as stem expands and ages.
none (1) = hairs appear to be absent from stems
few (2) = stem hairs are at low frequency
moderate (3) = stem hairs are at a regular and commonly seen density for most cotton cultivars
hairy (4) = stem hair density is greater than that normally seen on most cotton cultivars. Maybe essential for resistance to certain insects
very hairy (5) = highest density seen on stems. For cotton cultivars it likely has been selected for key loci to overexpress
pilose (6) = stem hairs are short and may lend velvety feel to leaf surfaces. Relative density is harder to describe and may be re-examined with digital images and/or supplemented in comments section. Best seen in *G. tomentosum* and some *G. barbadense*. Digital images are essential to confirm this trait in accessions which may have a variety of leaf hairs, differing in length.
seg/off type (9), rating to indicate that the plot is segregating for this trait and a single score is not an accurate description of the plot. Alternatively the rating is off the standard scale and best described as off type. Either is applicable to all the descriptors and best categorized this way and with same value (9). Comments may be added to clarify the multiple observations of this trait.

LEAF SIZE, numeric

The relative size of the mature, expanded leaf. Focus is on leaves from the fourth node from the growing tip. Digital images are taken of representative leaf on grid background for later application of metrics. In the field the size can be in relation to the hand.
medium (1) = leaf size is typical for that in cultivars, usually within the parameters of the opened adult hand.
small (2) = leaf size is less than the palm area of the opened adult hand.
large (3) = leaf size is greater than the opened adult hand, easily stretching past fingertips. Often *G. barbadense* accession or hybrids with this species have large leaf sizes
extra small (4) = leaf size is much smaller than that observed in cotton cultivars, but can be typical for some species. Best seen in *G. stocksii* and *G. armourianum*
seg/off type (9), rating to indicate that the plot is segregating for this trait and a single score is not an accurate description of the plot. Alternatively the rating is off the standard scale and best described as off type. Either is applicable to all the descriptors and best categorized this way and with same value (9). Comments may be added to clarify the multiple observations of this trait.

LEAF GLANDS, numeric

The relative density of gossypol glands in the leaves. These are observed as the small dark dots in the leaves. Gossypol glands impart chemical resistance to some herbivores and pests that consume the leaves
glandless (0) = no observable dark glands on the leaves. Most likely a result of intense selection against gossypol glands .
medium (1) = typically seen density of gossypol glands for most cotton cultivars.
light (2) = lower than normal density of gossypol glands. Likely was selected for alleles to underexpress.
heavy (3) = density of glands is greater than that normally seen in most cotton cultivars. Maybe a result of selection or is common for some species in the Genus, such as G. barbadense and G. arboreum.
seg/off type (9), rating to indicate that the plot is segregating for this trait and a single score is not an accurate description of the plot. Alternatively the rating is off the standard scale and best described as off type. Either is applicable to all the descriptors and best categorized this way and with same value (9). Comments may be added to clarify the multiple observations of this trait.

LEAF NECTARIES, alphanumeric

Commonly observed nectar glands on the undersides of the leaf. Numbers reflect their presence on one or more leaf veins. Nectar glands are nutritious to insects and microorganisms and may be a contributing factor to levels of insect damage or mold growth on plant and harvested cotton. Fully expanded leaves of each plant in the plot are observed to characterized the accession by the highest score observed because it is a trait that is highly variable on a single plant.
absent (0) = no nectar glands seen on the undersides of any leaves. Could be a result of negative selection or is typical of some species, e.g. *G. tomentosum*
one (1) = nectar glands only seed on the main vein of leaves
two (2) = nectar glands found on main vein and on one of the other veins
three (3) = nectar glands found on main vein and two other veins
four (4) = four nectar glands found on the leaf underside. Typically a second nectar gland is found on the main vein or on a smaller vein on a large leaf.
five (5) = five nectar glands found on the leaf underside. Typically nectar glands are found on the two more veins of a large leaf.
reduced (6) = nectar gland is barely noticeable on the main vein
seg/off type (9), rating to indicate that the plot is segregating for this trait and a single score is not an accurate description of the plot. Alternatively the rating is off the standard scale and best described as off type. Either is applicable to all the descriptors and best categorized this way and with same value (9). Comments may be added to clarify the multiple observations of this trait. Segregation for nectar glands is common in *G. arboreum* and *G. herbaceum*.

BRACT NECTARIES, numeric

The presence of nectar glands on the base of the bracts on the outside or abaxial side. It is absent in some species, e.g. *G. arboreum* and *G. herbaceum*. A flower or boll on each plant in the plot is observed to characterize the accession for presence, absence or segregation because it can be a trait that is variable on a single plant.
absent (0) = no nectar glands seen on base of bracts on the outside or abaxial side.
present (1) = nectar gland seen at least once on the plant on the outside or abaxial side.
reduced (2) = nectar gland is small in size and frequency on the plant.
inactive (3) = nectar is absent from the gland.
seg/off type (9), rating to indicate that the plot is segregating for this trait and a single score is not an accurate description of the plot. Alternatively the rating is off the standard scale and best described as off type. Either is applicable to all the descriptors and best categorized this way and with same value (9). Comments may be added to clarify the multiple observations of this trait. Segregation for nectar glands is common in *G. arboreum* and *G. herbaceum*.

BOLL NECTARIES, numeric

The presence of nectar glands on the base of the boll or calyx. It is particularly pronounced in some species, e.g. *G. arboreum*, *G. herbaceum*, and *G. barbadense*. A flower or boll on each plant in the plot is observed to characterize the accession for presence, absence or segregation because it can be a trait that is variable on a single plant.
absent (0) = no nectar glands seen on base of the boll.
present (1) = nectar gland seen at least once on the base of the boll.
reduced (2) = nectar gland is small in size and frequency on the plant.
inactive (3) = nectar is absent from the gland.
seg/off type (9), rating to indicate that the plot is segregating for this trait and a single score is not an accurate description of the plot. Alternatively the rating is off the standard scale and best described as off type. Either is applicable to all the descriptors and best categorized this way and with same value (9).

Comments may be added to clarify the multiple observations of this trait. Segregation for nectar glands is common in *G. arboreum* and *G. herbaceum*.

PETAL COLOR, numeric

Flower petal color
cream (1) = most common in cultivars of *G. hirsutum*.
yellow (2) = most common in *G. barbadense*, *G. herbaceum*, and *G. arboreum*.
light yellow (3) = seen in a few *G. barbadense* accessions such as those typed as "Braziliense".
red (4) = seen in a few *G. arboreum* and *G. hirsutum* ornamental cultivars.
white (5) = in a few G. arboreum accessions, typical for some species e.g. *G. costulatum*, *G. exiguum*, and *G. nobile*.
light blue (6) = in a few Australian diploids. Best observed in *G. sturtianum*.
golden (7) = *G. tomentosum*.
pink (8) = a few diploid species.
seg/off type (9), rating to indicate that the plot is segregating for this trait and a single score is not an accurate description of the plot. Alternatively the rating is off the standard scale and best described as off type. Either is applicable to all the descriptors and best categorized this way and with same value (9). Comments may be added to clarify the multiple observations of this trait.

POLLEN COLOR, numeric

Pollen color.
yellow (1) = seen in some of the older cultivars of *G. hirsutum*.
cream (2) = common in cultivars of *G. hirsutum*.
dark yellow (4) = typically seen in *G. barbadense*, *G. arboreum* and *G. herbaceum*.
orange (5) = extreme color, seen in some accessions of *G. barbadense*, considered a mutant.
seg/off type (9), rating to indicate that the plot is segregating for this trait and a single score is not an accurate description of the plot. Alternatively the rating is off the standard scale and best described as off type. Either is applicable to all the descriptors and best categorized this way and with same value (9). Comments may be added to clarify the multiple observations of this trait.

PETAL SPOT, numeric

Red coloration at base of flower petals, creating the effect of a spot in the center of the flower.
none (0) = base of petal is same color as rest of petal.
light (1) = red coloration is faint.
medium (2) = red coloration is just enough to create effect of petal spot.
heavy (3) = red coloration creates sold band across petals and extends halfway up the petal.
seg/off type (9), rating to indicate that the plot is segregating for this trait and a single score is not an accurate description of the plot. Alternatively the rating is off the standard scale and best described as off type. Either is applicable to all the descriptors and best categorized this way and with same value (9). Comments may be added to clarify the multiple observations of this trait.

STIGMA, numeric

The stigmatic column in relation to the antheridial cone.
normal (1) = stigma clears the antheridial cone. Typical for most *G. hirsutum* cultivars. Self pollinates easily.
protruding (2) = stigma is beyond the antheridial cone. Typical in many *G. barbadense* accessions and many species. Self pollination may be reduced particularly in greenhouses.
extreme protruding (3) = stigma is well beyond the antheridial cone and can surpass petal length. Self pollination must be done by hand and stigmas enclosed in bags to prevent outcrossing
short buried (4) = stigma is hidden by the tip of the antheridial cone.
seg/off type (9), rating to indicate that the plot is segregating for this trait and a single score is not an accurate description of the plot. Alternatively the rating is off the standard scale and best described as off type. Either is applicable to all the descriptors and best categorized this way and with same value (9). Comments may be added to clarify the multiple observations of this trait.

LINT COLOR, numeric

Color of mature fiber
white (1) = most cotton cultivars of *G. hirsutum* and *G. arboreum*.
cream (2) = many accessions of *G. barbadense* that show a slight yellowing of the mature fiber.
brown (3) = brown coloration of cotton producing species. Normal in *G. tomentosum*. Can darken with age.
green (4) = green coloration of fiber. Typically fades to off white or tan.
tan (5) = tan coloration of fiber.
rust (6) = strongest expression of brown and sometimes with hint of red. Best observed in rare, tropical accessions of G. barbadense.
off white (7) = best seen in *G. herbaceum* f. *africanum* and in some 'wild' accessions of cotton producing species that have smaller bolls and more sparse fiber.
seg/off type (9), rating to indicate that the plot is segregating for this trait and a single score is not an accurate description of the plot. Alternatively the rating is off the standard scale and best described as off type. Either is applicable to all the descriptors and best categorized this way and with same value (9). Comments may be added to clarify the multiple observations of this trait.

LOCULE NUMBER, numeric

Number of locules per boll, determined by selecting the mode or the whole number nearest the average value. It basically indicates how many places the boll split to reveal the seed
1 (1) = one locule, a highly unlikely value.
2 (2) = two locules.
3 (3) = three locules.
4 (4) = four locules.
5 (5) = five locules.
>5 (6) = greater than five locules per boll.
seg/off type (9), rating to indicate that the plot is segregating for this trait and a single score is not an accurate description of the plot. Alternatively the rating is off the standard scale and best described as off type. Either is applicable to all the descriptors and best categorized this way and with same value (9). Comments may be added to clarify the multiple observations of this trait.

SEED FUZZ, numeric

The relative density of seed fuzz on the seed surface. Best observed after ginning the cotton. Hand or roller ginning, if possible gives the clearest look at the seed fuzz
none (0) = no seed fuzz on surface.

medium (1) = seed fuzz throughout surface.
high (2) = dense seed fuzz and seed coat is not visible.
sparse (3) = seed fuzz occurs sparingly on surface.
tufted (4) = fuzz only on micropylar end.
seg/off type (9), rating to indicate that the plot is segregating for this trait and a single score is not an accurate description of the plot. Alternatively the rating is off the standard scale and best described as off type. Either is applicable to all the descriptors and best categorized this way and with same value (9). Comments may be added to clarify the multiple observations of this trait.

SEED FUZZ COLOR, numeric

The color of the seed fuzz. Best observed after ginning. Hand or roller ginning, if possible, gives the clearest look at the seed fuzz color.
lintless (0) = no fuzz or lint on seed surface.
white (1) = white colored seed fuzz.
cream (2) = cream colored seed fuzz.
brown (3) = brown colored seed fuzz.
green (4) = green colored seed fuzz. Maybe be a brighter green color than what is typically seen in green fiber color. It eventually fades to tan or brown.
tan (5) = tan colored seed fuzz.
rust (6) = darkest color of fuzz on the seed.
off white (7) = off white colored seed fuzz.
seg/off type (9), rating to indicate that the plot is segregating for this trait and a single score is not an accurate description of the plot. Alternatively the rating is off the standard scale and best described as off type. Either is applicable to all the descriptors and best categorized this way and with same value (9). Comments may be added to clarify the multiple observations of this trait.

SEED TYPE, numeric

The clustering of the seed in the boll.
free (1) = seeds separate freely from each other in the ripe boll.
semi kidney (2) = some seeds remain stuck to each other even after boll is fully ripe.
kidney (3) = seeds are stuck together in the ripe boll. Typically in some *G. barbadense* accession typed as "Braziliense".
seg/off type (9), rating to indicate that the plot is segregating for this trait and a single score is not an accurate description of the plot. Alternatively the rating is off the standard scale and best described as off type. Either is applicable to all the descriptors and best categorized this way and with same value (9). Comments may be added to clarify the multiple observations of this trait.

BRACT TYPE, numeric

Type of bract according to shape or placement relative to the boll.
normal (1) = typical bract that remains close to the boll and expands to partially wrap the boll. For lack of a better descriptor in some exotic species it is the wild type.
frego (2) = bract is reduced in overall growth. Less likely to contribute to trash on harvest cotton.
flared (3) = bract tends to point away from the boll. Best obsevered on *G. herbaceum* f. *africanum*.
recurved (4) = bract tends to point further away from the boll towards the pedicel.
seg/off type (9), rating to indicate that the plot is segregating for this trait and a single score is not an accurate description of the plot. Alternatively the rating is off the standard scale and best described as off type. Either is applicable to all the descriptors and best categorized this way and with same value (9). Comments may be added to clarify the multiple observations of this trait.

BRACT TEETH SIZE, numeric

Size of bract teeth relative to the bract. Deeply incised teeth generate more fine trash on harvest cotton.
none (0) = bract margins are smooth with no teeth.
medium (1) = teeth indent to about 1/3 the overall length of the bract.
small (2) = teeth make little indentation into bract length. Found often on *G. arboreum*.
large (3) = bracts are indented to half of length of the bract. Common for *G. hirsutum* cultivars.
seg/off type (9), rating to indicate that the plot is segregating for this trait and a single score is not an accurate description of the plot. Alternatively the rating is off the standard scale and best described as off type. Either is applicable to all the descriptors and best categorized this way and with same value (9). Comments may be added to clarify the multiple observations of this trait.

BRACT TEETH NUMBER, numeric

Relative number of bract teeth to what is typically seen for cultivars of *G. hirsutum*.
medium (1) = around a dozen teeth on the bract.
many (2) = more than a dozen teeth on the bract. Best observed on *G. raimondii*.
few (3) = less than a dozen teeth on the bract. Typical for many species.
seg/off type (9), rating to indicate that the plot is segregating for this trait and a single score is not an accurate description of the plot. Alternatively the rating is off the standard scale and best described as off type. Either is applicable to all the descriptors and best categorized this way and with same value (9). Comments may be added to clarify the multiple observations of this trait.

BRACT COLOR, numeric

The degree of red coloration to the bracts. Red coloration has value as an ornamental, marker plant or possibly to resist certain insects.
green (1) = green colored bracts.
red (2) = solid red coloration of the bracts.
sun red (3) = partial red color of the bracts. Generally the norm and difficult to tell from stress.
seg/off type (9), rating to indicate that the plot is segregating for this trait and a single score is not an accurate description of the plot. Alternatively the rating is off the standard scale and best described as off type. Either is applicable to all the descriptors and best categorized this way and with same value (9). Comments may be added to clarify the multiple observations of this trait.

BOLL SHAPE, numeric

General description of degree of roundness to the boll shapes.
oval (1) = oval shape to bolls. Widest point tends to the middle of the boll.
round (2) = round shape to the bolls. Not necessarily perfectly round but length is close to width.

cone (3) = bolls are wider at the base and overall shape tapers to the tip. Common in *G. barbadense*.
cone oval (4) = bolls show tapering from middle to the tip. The base and middle are comparable in length. Common in *G. arboreum*.
seg/off type (9), rating to indicate that the plot is segregating for this trait and a single score is not an accurate description of the plot. Alternatively the rating is off the standard scale and best described as off type. Either is applicable to all the descriptors and best categorized this way and with same value (9). Comments may be added to clarify the multiple observations of this trait.

BOLL POINT, numeric

Degree of tapering of the boll tips.
moderately pointed (1) = boll ends in a point, intermediate between clearly pointed and blunt.
pointed (2) = boll is tapered to a tip. It can complement the cone boll shape or appear pinched out from the oval and round bolls.
blunt (3) = boll ends abruptly. With round or oval bolls, tip follows the overall shape of the boll.
seg/off type (9), rating to indicate that the plot is segregating for this trait and a single score is not an accurate description of the plot. Alternatively the rating is off the standard scale and best described as off type. Either is applicable to all the descriptors and best categorized this way and with same value (9). Comments may be added to clarify the multiple observations of this trait.

BOLL SIZE, numeric

Relative size of the bolls to what is typical for most *G. hisutum* cultivars (golf ball size for mature but non cleft bolls).
medium (1) = typical size for most cotton cultivars (golf ball size).
large (2) = larger than typical boll size, larger than golf ball size. May also be longer than normal for *G. barbadense*.
small (3) = smaller than typical size in most cotton cultivars. Best seen in a number of *G. herbaceum* accessions.
extra small (4) = typical for most of the exotic species, think marble size or smaller.
seg/off type (9), rating to indicate that the plot is segregating for this trait and a single score is not an accurate description of the plot. Alternatively the rating is off the standard scale and best described as off type. Either is applicable to all the descriptors and best categorized this way and with same value (9). Comments may be added to clarify the multiple observations of this trait.

BOLL COLOR, numeric

The degree of red coloration to the boll. Red coloration has value as an ornamental, marker plant or possibly to resist certain insects.
green (1) = typical for most cotton cultivars.
dark green (2) = darker color than most of the cotton cultivars. Often seen in *G. barbadense*.
light green (3) = lighter color than most of the cotton cultivars. Often seen in *G. arboreum*.
red (4) = red coloration to the boll color.
sun red (5) = red coloration to the boll color in areas exposed to the sun. Typically green shade marks are left by the bracts.
seg/off type (9), rating to indicate that the plot is segregating for this trait and a single score is not an accurate description of the plot. Alternatively the rating is off the standard scale and best described as off type. Either is applicable to all the descriptors and best categorized this way and with same value (9). Comments may be added to clarify the multiple observations of this trait.

BOLL GLANDING, numeric

Relative density of gossypol glands on the surface of the mature, but non cleft boll. These are observed as the small dark dots on the stems. Gossypol glands impart resistance to some herbivores and pests, but complicate purification of oils and protein from seeds.
glandless (0) = no observable gossypol glands on the boll surface.
medium (1) = typical density of gossypol glands observed on most *G. hirsutum* cultivars.
light (2) = less gossypol glands than is normally observed on most *G. hirsutum* cultivars.
heavy (3) = more gossypol glands than is normally observed on most cotton cultivars. Often seen with *G. barbadense* and *G. arboreum* accessions.
seg/off type (9), rating to indicate that the plot is segregating for this trait and a single score is not an accurate description of the plot. Alternatively, the rating is off the standard scale and best described as off type. Either is applicable to all the descriptors and best categorized this way and with same value (9). Comments may be added to clarify the multiple observations of this trait.

BOLL PITTING, numeric

Relative intensity of the pitting of the surface of the mature, but non cleft bolls.
smooth (1) = no observable pitting of the surface of the boll
lightly pitted (2) = low degree of pitting, shallower pits and lower density than types representative of boll pitting (e.g *G. barbadense* and *G. arboreum*)
pitted (3) = typical degree of pittin of the boll surface and common in *G. barbadense* and *G. arboreum*
very pitted (4) = intense pitting of boll surface in density and depth. Seen on some *G. barbadense* and *G. arboreum*
seg/off type (9), rating to indicate that the plot is segregating for this trait and a single score is not an accurate description of the plot. Alternatively, the rating is off the standard scale and best described as off type. Either is applicable to all the descriptors and best categorized this way and with same value (9). Comments may be added to clarify the multiple observations of this trait.

FRUITING TYPE, numeric

Pattern of boll formation on branches. Known to be affected by specific alleles in cotton.
normal (1) = regular formation of bolls, with 1-2 per node. In the absence of a better descriptor for most species of *Gossypium* it is the wild type.
cluster (2) = bolls are formed on prematurely shortened branches and sometimes only on the stem. Three bolls may form at the end of the truncated branch.
seg/off type (9), rating to indicate that the plot is segregating for this trait and a single score is not an accurate description of the plot. Alternatively, the rating is off the standard scale and best described as off type. Either is applicable to all the descriptors and best categorized this way and with same value (9). Comments may be added to clarify the multiple observations of this trait.
