# Supplementary material for: Leveraging National Germplasm Collections to Determine Significantly Associated Categorical Traits in Crops: Upland and Pima Cotton as a Case Study
Source: Front Plant Sci. 2022 Apr 26;13:837038. doi: 10.3389/fpls.2022.837038 (PMC9087864; doi:10.3389/fpls.2022.837038)
Supplement: Supplementary Data 2 — “One_hot-ordinal.py” script to transform data to apply the clustering analysis. [file Data_Sheet_2.PDF]

```
#!/usr/bin/env python2
# -*- coding: utf-8 -*-
"""
Created on Wed Oct 16 17:30:26 2019
@author: drestre
Data S2 - one_hot-ordinal encoding
"""
import pandas as pd
#import numpy as np
from sklearn.preprocessing import OneHotEncoder
from sklearn.preprocessing import OrdinalEncoder
from kmodes.kmodes import KModes
from sklearn.metrics import silhouette_score
#import matplotlib.pyplot as plt

'''
Range of Silhouette Value -

Now, S(i) will lie between [-1, 1] -

1. If silhouette value is close to 1, sample is well-clustered and already
assigned to a very appropriate cluster.

2. If silhouette value is about to 0, sample could be assign to another
cluster closest to it and the sample lies equally far away from both the
clusters. That means it indicates overlapping clusters

3. If silhouette value is close to -1, sample is misclassified and is
merely
placed somewhere in between the clusters.
'''

df = pd.read_csv("1297_sa_tx_gb_proof_v1.csv", header=0, sep='\t')

########### ordinal enconder - OrdinalEncoder - sklearn ############
#example:
#encoder_leafhair = OrdinalEncoder(categories=['none', 'some', 'pilose'])
#cat_leafhair = pd.Categorical(df.leafhair, categories=['none', 'some',
#                                                       'pilose'],
ordered=True)
#labels, unique = pd.factorize(cat_leafhair, sort=True)
#df.leafhair = labels

##########

encoder_leafhair = OrdinalEncoder(categories=['segofftype', 'none', 'few',
'moderate', 'hairy', 'veryhairy', 'pilose'])
cat_leafhair = pd.Categorical(df.leafhair, categories=['segofftype',
```

```
'none', 'few', 'moderate', 'hairy', 'veryhairy', 'pilose'])
labels, unique = pd.factorize(cat_leafhair, sort=True)
df.leafhair = labels

encoder_stemglands = OrdinalEncoder(categories=['segofftype', 'glandless',
'light', 'medium', 'heavy'])
cat_stemglands = pd.Categorical(df.stemglands, categories=['segofftype',
'glandless', 'light', 'medium', 'heavy'])
labels, unique = pd.factorize(cat_stemglands, sort=True)
df.stemglands = labels

enconder_stemhair = OrdinalEncoder(categories=['segofftype', 'none', 'few',
'moderate', 'hairy', 'veryhairy', 'pilose'])
cat_stemhair = pd.Categorical(df.stemhair, categories=['segofftype',
'none', 'few', 'moderate', 'hairy', 'veryhairy', 'pilose'])
labels, unique = pd.factorize(cat_stemhair, sort=True)
df.stemhair = labels

encoder_leafsize = OrdinalEncoder(categories=['segofftype', 'extrasmall',
'small', 'medium', 'large'])
cat_leafsize = pd.Categorical(df.leafsize, categories=['segofftype',
'extrasmall', 'small', 'medium', 'large'])
labels, unique = pd.factorize(cat_leafsize, sort=True)
df.leafsize = labels

encoder_leafglands = OrdinalEncoder(categories=['segofftype', 'glandless',
'light', 'medium', 'heavy'])
cat_leafglands = pd.Categorical(df.leafglands, categories=['segofftype',
'glandless', 'light', 'medium', 'heavy'])
labels, unique = pd.factorize(cat_leafglands, sort=True)
df.leafglands = labels

encoder_leafnectaries = OrdinalEncoder(categories=['segofftype', 'absent',
'reduced', 'mainvein', 'two', 'three', 'four'])
cat_leafnectaries = pd.Categorical(df.leafnectaries,
categories=['segofftype', 'absent', 'reduced', 'mainvein', 'two', 'three',
'four'])
labels, unique = pd.factorize(cat_leafnectaries, sort=True)
df.leafnectaries = labels

encoder_petalspot = OrdinalEncoder(categories=['segofftype', 'none',
'light', 'medium', 'heavy'])
cat_petalspot = pd.Categorical(df.petalspot, categories=['segofftype',
'none', 'light', 'medium', 'heavy'])
labels, unique = pd.factorize(cat_petalspot, sort=True)
df.petalspot = labels

encoder_stigma = OrdinalEncoder(categories=['segofftype', 'normal',
```

```
79… 'protruding', 'extremeprotruding'])
80  cat_stigma = pd.Categorical(df.stigma, categories=['segofftype', 'normal',
…   'protruding', 'extremeprotruding'])
81  labels, unique = pd.factorize(cat_stigma, sort=True)
82  df.stigma = labels
83
84  encoder_loculenum = OrdinalEncoder(categories=['segofftype', 'three',
…   'four', 'five'])
85  cat_loculenum = pd.Categorical(df.loculenum, categories=['segofftype',
…   'three', 'four', 'five'])
86  labels, unique = pd.factorize(cat_loculenum, sort=True)
87  df.loculenum = labels
88
89  encoder_seedfuzz = OrdinalEncoder(categories=['segofftype', 'none',
…   'tufted', 'medium', 'sparse', 'high'])
90  cat_seedfuzz = pd.Categorical(df.seedfuzz, categories=['segofftype',
…   'none', 'tufted', 'medium', 'sparse', 'high'])
91  labels, unique = pd.factorize(cat_seedfuzz, sort=True)
92  df.seedfuzz = labels
93
94  encoder_bractteethsize = OrdinalEncoder(categories=['segofftype', 'small',
…   'medium', 'large'])
95  cat_bractteethsize = pd.Categorical(df.bractteethsize,
…   categories=['segofftype', 'small', 'medium', 'large'])
96  labels, unique = pd.factorize(cat_bractteethsize, sort=True)
97  df.bractteethsize = labels
98
99  encoder_bractteethnumber = OrdinalEncoder(categories=['segofftype', 'few',
…   'medium', 'many'])
100 cat_bractteethnumber = pd.Categorical(df.bractteethnumber,
…   categories=['segofftype', 'few', 'medium', 'many'])
101 labels, unique = pd.factorize(cat_bractteethnumber, sort=True)
102 df.bractteethnumber = labels
103
104 encoder_bollpoint = OrdinalEncoder(categories=['segofftype', 'blunt',
…   'moderatelypointed', 'pointed'])
105 cat_bollpoint = pd.Categorical(df.bollpoint, categories=['segofftype',
…   'blunt', 'moderatelypointed', 'pointed'])
106 labels, unique = pd.factorize(cat_bollpoint, sort=True)
107 df.bollpoint = labels
108
109 encoder_bollsize = OrdinalEncoder(categories=['segofftype', 'extrasmall',
…   'small', 'medium', 'large'])
110 cat_bollsize = pd.Categorical(df.bollsize, categories=['small', 'medium',
…   'large'])
111 labels, unique = pd.factorize(cat_bollsize, sort=True)
112 df.bollsize = labels
113
```

```
encoder_bollglanding = OrdinalEncoder(categories=['segofftype',
… 'glandless', 'light', 'medium', 'heavy'])
cat_bollglanding = pd.Categorical(df.bollglanding,
… categories=['segofftype', 'glandless', 'light', 'medium', 'heavy'])
labels, unique = pd.factorize(cat_bollglanding, sort=True)
df.bollglanding = labels

encoder_bollpitting = OrdinalEncoder(categories=['segofftype', 'smooth',
… 'lightlypitted', 'pitted', 'verypitted'])
cat_bollpitting = pd.Categorical(df.bollpitting, categories=['segofftype',
… 'smooth', 'lightlypitted', 'pitted', 'verypitted'])
labels, unique = pd.factorize(cat_bollpitting, sort=True)
df.bollpitting = labels

############ nominal encoder - OneHotEnconder - sklearn ############
#onehot = OneHotEncoder(dtype=np.int, sparse=True)
#nominals = pd.DataFrame(onehot.fit_transform(df[['growthhabit',
… 'canopy_type',
#                                                 'leafcolor', 'leafshape',
#                                                 'stemcolor',
… 'bractnectaries',
#                                                 'bollnectaries',
… 'petalcolor',
#                                                 'pollencolor',
#
]]).toarray(), \
#                         columns=['normal', 'spreading', 'pyramid',
… 'stovepipe',
#                                  'seg/offtype', 'typical', 'open',
… 'dense',
#                                  'compact', 'segofftype', 'green', 'red',
#                                  'darkred', 'seg/offtype', 'normal',
… 'okra',
#                                  'subokra', 'superokra', 'seg/offtype',
#                                  'green', 'sunred', 'red', 'seg/offtype',
#                                  'absent', 'present', 'reduced',
… 'seg/offtype',
#                                  'absent', 'present', 'reduced',
… 'seg/offtype',
#                                  'cream', 'yellow', 'lightyellow', 'red',
#                                  'seg/offtype', 'intermed', 'yellow',
… 'cream',
#                                  'seg/offtype', 'darkyellow', 'orange'])

columns_to_encode = ['growthhabit', 'growthhabit', 'canopy_type',
… 'leafcolor',
                     'leafshape', 'stemcolor', 'bractnectaries',
                     'bollnectaries', 'petalcolor', 'pollencolor',
```

```
'lintcolor',
                      'seedfuzzcolor', 'seedtype', 'bracttype',
'bractcolor',
                      'bollshape', 'bollcolor', 'fruitingtype']
ohe = OneHotEncoder(sparse=False)
encoded_columns = ohe.fit_transform(df[columns_to_encode])
dataframe = pd.DataFrame(encoded_columns)

# coherse dataframe nomila ordinal - following oridinal df order
dataframe['leafhair'] = df.leafhair
dataframe['stemglands'] = df.stemglands
dataframe['stemhair'] = df.stemhair
dataframe['leafsize'] = df.leafsize
dataframe['leafglands'] = df.leafglands
dataframe['leafnectaries'] = df.leafnectaries
dataframe['petalspot'] = df.petalspot
dataframe['stigma'] = df.stigma
dataframe['loculenum'] = df.loculenum
dataframe['seedfuzz'] = df.seedfuzz
dataframe['bractteethsize'] = df.bractteethsize
dataframe['bractteethnumber'] = df.bractteethnumber
dataframe['bollpoint'] = df.bollpoint
dataframe['bollsize'] = df.bollsize
dataframe['bollglanding'] = df.bollglanding
dataframe['bollpitting'] = df.bollpitting

#nominals['leafhair'] = df.leafhair
#nominals['stemglands'] = df.stemglands
#nominals['stemhair'] = df.stemhair
#nominals['leafsize'] = df.leafsize
#nominals['leafglands'] = df.leafglands
#nominals['leafnectaries'] = df.leafnectaries

#nominals['canopy/type'] = df.canopy_type

#columns_to_encode = ['growthhabit']
#ohe = OneHotEncoder(sparse=False)
#encoded_columns = ohe.fit_transform(df[columns_to_encode])


#columns_to_encode = ['growthhabit', 'leafhair', 'bractteethsize']

#scaler = StandardScaler()
#ohe = OneHotEncoder(sparse=False)

#scaled_columns = scaler.fit_transform(df[columns_to_scale])
#encoded_columns = ohe.fit_transform(df[columns_to_encode])

```

```
range_n_clusters = [3]

for n_clusters in range_n_clusters:
    #create plot
    #fig, (ax1, ax2) = plt.subplots(1, 2)
    #fig.set_size_inches(18, 7)

    #1rst subplot is the silhouette plot range -1/1
    #ax1.set_xlim([-0.1, 1])
    #The (n_clusters+1)*10 is for inserting blanck space between silhouette
    #ax1.set_ylim([0, len(encoded_columns) + (n_clusters + 1) * 100])

    #KModes
    km = KModes(n_clusters=n_clusters, init='Huang', n_init=100, verbose=1)
    clusters = km.fit_predict(dataframe)

    #The silhouette_score

    silhouette_avg = silhouette_score(dataframe, clusters)
    print ("For no of clusters =", n_clusters,
           "The average silhouette_score is: ", silhouette_avg)
```
