# Supplementary material for: Leveraging National Germplasm Collections to Determine Significantly Associated Categorical Traits in Crops: Upland and Pima Cotton as a Case Study
Source: Front Plant Sci. 2022 Apr 26;13:837038. doi: 10.3389/fpls.2022.837038 (PMC9087864; doi:10.3389/fpls.2022.837038)
Supplement: Supplementary Data 3 — “MCA_ind_corr_contr_sa_tex_gb.R” script to calculate MCA and plot results. [file Data_Sheet_3.PDF]

```r
library("FactoMineR")
library("factoextra")
library("ggplot2")

###### options
options(ggrepel.max.overlaps = 17) # set number of tags 10 equals default, inf equals all
options(scipen = 999) #tun of scientific notation / options(scipen = 0) # revert by default
######
# this analysis includes SA, TEX, and GB classes - the dataframe includes frequency
my.data.famd <- read.csv("Table_S7_new_noids.csv",
                         header = TRUE,
                         sep  = ";"
                         )
res.famd <- FAMD(my.data.famd, #MCA by traits-states, FAMD by traits, MFA
                 graph = FALSE,
                 #ind.sup = "IDENT",
                 ncp = 10
                 ) # it was TRUE for Table_S7_new.csv false for noids
# MFA visualization library using the MCA results
data.mfa.sa.tex.gb <- fviz_mfa_ind(res.famd,
                                   habillage = "IDENT", # color by groups
                                   #"quali.var",
                                   geom = c("point", "text"), # text, point, arrow
                                   #shape.ind = 1, # 5 rombus, 2 triang, 1 empty cir, 6 trian
                                   #pointsize = my.data.frame.index, #use request col as pointsize
                                   palette = c("#00AFBB", "#E7B800", "#FC4E07"),
                                   addEllipses = FALSE, ellipse.type = "confidence",
                                   repel = TRUE, # Avoid text overlapping
                                   title = "Multiple Correspondence Analysis of descriptors to first 2 component dimension of SA, TEX, and GB",
                                   )

# This analysis includes a dataframe without ids
my.data.famd.noids.nofreq <- read.csv("Table_S7_new_noids_nofreq.csv",
                                      header = TRUE,
                                      sep  = ";"
                                      )
res.famd.noid.nofreq <- FAMD(my.data.famd.noids.nofreq,
                             graph = FALSE)

```

```r
data.corr.sa.sa.tex.gb <- fviz_famd_var(res.famd.noid.nofreq,
                                        repel = TRUE,
                                        graph = TRUE, #correlation between variables
                                        title = "Correlation of descriptors to first 2 component dimensions for SA, TEX, and GB" #correlation between variables
                                        )
data.contrib.sa.tex.gb <- fviz_contrib(res.famd.noid.nofreq,
                                      "var",
                                      axes = 1:2,
                                      title = "Contribution of descriptors to first 2 component dimensions for SA, TEX, and GB"
                                      ) #contribution
#data.contrib.sa.tex.gb$eig

####### SA- MCA results
my.data.famd.sa <- read.csv("Table_S7_SA.csv",
                            header = TRUE,
                            sep  = ";"
                            )
res.famd.sa <- FAMD(my.data.famd.sa,
                    graph = FALSE
                    )
#print(res.famd.sa)

# SA - Correlation and contribution plots
data.corr.sa <- fviz_famd_var(res.famd.sa,
                              repel = TRUE,
                              graph = TRUE,
                              title = "Correlation of descriptors to first 2 component dimensions of SA" #correlation between variables
                              )

data.contrib.sa <- fviz_contrib(res.famd.sa,
                               "var",
                               axes = 1:2, #contribution
                               title = "Contribution of descriptors to first 2 component dimensions of SA"
                               )
######## TEX- MCA results
my.data.famd.tex <- read.csv("Table_S7_TEX.csv",
                             header = TRUE,
                             sep  = ";"
                             )
res.famd.tex <- FAMD(my.data.famd.tex,
                     graph = FALSE
                     )
```

```r
print(res.famd.tex)

# TEX - Correlation and contribution plots
data.corr.tex <- fviz_famd_var(res.famd.tex,
                               repel = TRUE,
                               graph = TRUE,
                               title = "Correlation of descriptors to first 2 component dimensions of TEX" #correlation between variables
                               )

data.contrib.tex <- fviz_contrib(res.famd.tex,
                                 "var",
                                 axes = 1:2,
                                 title = "Contribution of descriptors to first 2 component dimensions of TEX"
                                 )
######## GB - MCA results
my.data.famd.gb <- read.csv("Table_S7_GB.csv",
                            header = TRUE,
                            sep  = ";"
                            )

res.famd.gb <- FAMD(my.data.famd.gb,
                    graph = FALSE
                    )
#print(res.famd.gb)
# GB- Correlation and contribution plots
data.corr.gb <- fviz_famd_var(res.famd.gb,
                              repel = TRUE,
                              graph = TRUE,
                              title = "Correlation of descriptors to first 2 component dimensions of GB"
                              ) #correlation between variables

data.contrib.gb <- fviz_contrib(res.famd.gb,
                                "var",
                                top = Inf,
                                axes = 1:2,
                                title = "Contribution of descriptors to first 2 component dimensions of GB"
                                ) #contribution

fviz_eig(res.famd.gb, addlabels = TRUE)
```
